# Hydrogen Production from Gadolinium-Promoted Yttrium-Zirconium-Supported Ni Catalysts through Dry Methane Reforming

**DOI:** 10.1021/acsomega.3c02229

**Published:** 2023-06-08

**Authors:** Anis H. Fakeeha, Ahmed S. Al-Fatesh, Vijay Kumar Srivastava, Ahmed A. Ibrahim, Abdulaziz A.M. Abahussain, Jehad K. Abu-Dahrieh, Mohammed F. Alotibi, Rawesh Kumar

**Affiliations:** † Chemical Engineering Department, College of Engineering, King Saud University, P.O. Box 800, Riyadh 11421, Saudi Arabia; ‡ Department of Chemistry, Indus University, Ahmedabad, Gujarat 382115, India; § School of Chemistry and Chemical Engineering, Queen’s University Belfast, Belfast BT9 5AG, Northern Ireland, UK; ∥ Institute of Refining and Petrochemicals Technologies, King Abdulaziz City for Science and Technology (KACST), P.O. Box 6086, Riyadh 11442, Kingdom of Saudi Arabia

## Abstract

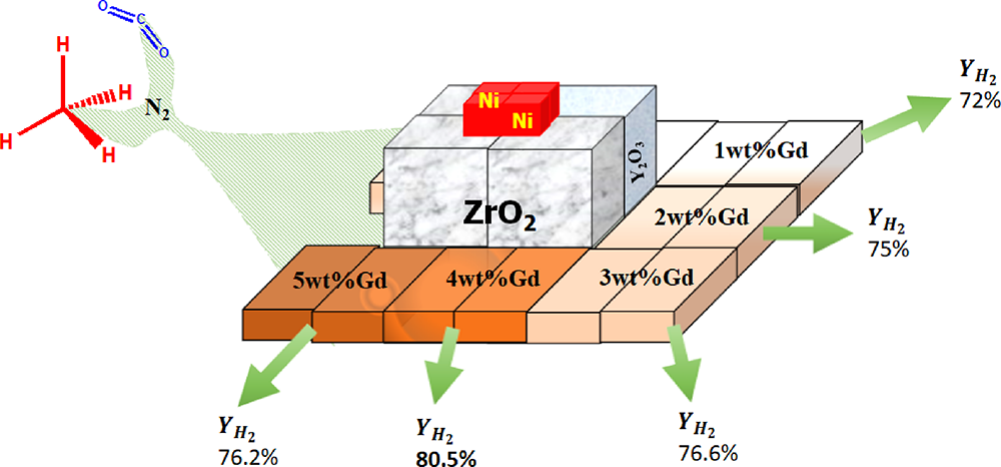

Hydrogen production from dry reforming of methane (DRM) not only concerns with green energy but also involves the consumption of two greenhouse gases CH_4_ and CO_2_. The lattice oxygen endowing capacity, thermostability, and efficient anchoring of Ni has brought the attention of the DRM community over the yttria-zirconia-supported Ni system (Ni/Y + Zr). Herein, Gd-promoted Ni/Y + Zr is characterized and investigated for hydrogen production through DRM. The H_2_-TPR → CO_2_-TPD → H_2_-TPR cyclic experiment indicates that most of the catalytic active site (Ni) remains present during the DRM reaction over all catalyst systems. Upon Y addition, the tetragonal zirconia-yttrium oxide phase stabilizes the support. Gadolinium promotional addition up to 4 wt % modifies the surface by formation of the cubic zirconium gadolinium oxide phase, limits the size of NiO, and makes reducible NiO moderately interacted species available over the catalyst surface and resists coke deposition. The 5Ni4Gd/Y + Zr catalyst shows about ∼80% yield of hydrogen constantly up to 24 h at 800 °C.

## 1 Introduction

The green routes acclaimed for hydrogen production concern with electrolysis of water, steam reforming of methane (H_2_/CO ∼3), partial oxidation of methane (H_2_/CO ∼2), and dry reforming of methane (DRM) (H_2_/CO ∼1).^1^ Although the H_2_/CO ratio of DRM is low, it has drawn huge attention because by this route, two global warming gases (CH_4_ and CO_2_) are precisely converted. Therefore, it bears the hope of reduction of the concentration of global warming gases. Additionally, this route generates energy prime hydrogen-rich syngas.

DRM reaction is a highly endothermic reaction, and it is catalyzed by different active metals Pt, Pd, Ru, Co, and Ni. The methane dissociation energy is found in the order Ni < Pd = Pt.^2^ Noble metals show better activity, but they are extremely expensive; a good replacement would be nickel, which has shown the best activity among transition metals. The methane interaction energy over Ni is 18 kcal/mol, which is 25 times higher than that of Co (methane interaction energy over Co is 0.7 kcal/mol).^3^ However, Ni is highly unstable at high temperatures where it is sintered into large sizes, stimulates large coke deposition, and finally ends with deactivation. Silica, alumina, aluminosilicate, and zirconia supports have a high metal–support interaction with Ni, and so Ni particles are held over these supports even against very high temperatures. Silica was used as a neutral support, alumino-silicate support offered acidity at a high Si/Al ratio,^4^ and alumina support was known to provide acidity to the catalyst system.^5^ Promotional addition of ZrO_2_ over an Al_2_O_3_-supported Ni catalyst was found to increase Ni dispersion and decrease coke deposition.^6,7^ Zhang et al. carried out CH_4_-temperature-programmed surface reaction over Ni/ZrO_2_ and detected CO.^8^ This observation indicates the involvement of lattice oxygen of ZrO_2_ in carbon deposit oxidation. Therefore, ZrO_2_ can endow lattice oxygen, withstand high-temperature conditions, and provide an efficient Ni anchoring effect by Zr^+4^.

A bimetallic Ni-Co particle dispersion over the ZrO_2_-MgO (9:1) support was found effective in H_2_ yield (85%) in the presence of 7 mol % oxygen along with a 3:1 CO_2_ and CH_4_ ratio.^9^ However, in DRM, CO_2_ is a promising oxidizing agent for oxidation of CH_4_ and use of oxygen is completely restricted. Furthermore, “Ni impregnated over ZrO_2_” was employed by different groups for DRM but due to instability of ZrO_2_ phases against high temperature, H_2_ yield never exceeded more than 50%.^10−12^ Silica-promoted ZrO_2_-supported Ni catalysts have brought attention due to 1.5% H_2_ yield up to 15 h at a very low temperature (400 °C) compared to the conventional 700–800 °C reaction temperature.^13^ ZrO_2_-rich ceria (28 wt %)-supported Ni catalysts had better stability under a thermally reductive environment than individual metal oxide-supported Ni catalysts.^14^ They also induced Ni dispersion and gave 35% H_2_ yield up to a 24 h time on stream (TOS) at a 700 °C reaction temperature^14^ and about ∼40% H_2_ yield at 750 °C for 50 h.^15^ The presence of ceria along with ZrO_2_ had a stabilizing effect on tetragonal ZrO_2_, enhancing lattice oxygen mobility and increasing reducibility in favor of DRM and ceria-zirconia-supported Ni catalysts, conveying more than 57% H_2_ yield.^16−18^ Upon 0.02 mol Ca incorporation with 0.04 mol Ni and 0.1 mol ZrO_2_ (via reflux-mediated coprecipitation), both the surface parameter and the metal–surface interaction were enhanced and about 65% H_2_ yield was obtained.^10^ Lanthana addition in the ZrO_2_ support brought stability of the tetragonal phase of ZrO_2_ and formation of La_2_O_2_CO_3_ species for efficient removal of carbon deposit. A 10 wt % lanthana–90 wt % ZrO_2_-supported Ni catalyst showed 74% H_2_ yield up to 400 min.^19^ Further promotional addition of basic Ca over a lanthana-zirconia-supported system brought a more efficient CO_2_ interaction, and the Ni–O-Ca interphase helped Ni to restore Ni particles to the original state^20^ for efficient CH_4_ decomposition. It brought interest in low-temperature DRM reaction as it showed ∼6.1% H_2_ yield at a 450 °C reaction temperature after 30 min of reaction. Ceria promotional addition over lanthana-zirconia-supported nickel reduced the band gap and added additional lattice oxygen mobility, prominent CH_4_ decomposition sites, resistance of ZrO_2_ phase transition, and prominent interaction of CO_2_.^11^ It gave 75% H_2_ yield at 460 min TOS. A chromium-promoted lanthana-zirconia-supported system stabilized the tetragonal ZrO_2_ phase, stabilized the lanthana-zirconia phase, and showed excellent oxygen replenishment capacity as reduced NiO was re-oxidized by CO_2_ up to the optimum level. It showed about 80% H_2_ yield.^21^ The promotional addition of Ce over the tungsten-zirconia support also had a stable tetragonal zirconia phase.^22^ It had additional basic sites, a ceria tungsten oxide phase for instant release of oxygen, and an excellent re-oxidizing capacity of reducible NiO. It resulted in 78% H_2_ yield at 420 min TOS. Y addition with zirconia was found to stabilize^12^ tetragonal ZrO_2_ as well as facilitate additional O^2–^ species in the lattice. A 15 wt % yttria–85 wt % zirconia-supported Ni catalyst had a wide range of basic sites, and it showed 78% H_2_ yield. A nonmetal oxide–metal oxide-supported Ni catalyst, as well as nonmetal-promoted DRM catalyst systems, was also tried; however, we are limiting our literature to metal-promoted and binary metal oxide-supported DRM catalysts.^23,24^

Gd was used as a spacer over iron oxide-based catalysts, which caused the increase in specific area. Its presence inhibited the reduction of Fe^+3^ to Fe^+2^ and the formation of iron carbide.^25^ Gadolinium ferrites had p-type conductivity, which favors the chemisorption of CO molecules.^39−41^ Gadolinium-doped ceria depressed the bulk but increased the dynamic oxygen exchange capacity (OEC).^26,27^ There is a strong 3d–4f electron exchange, and a spin–orbit interaction is noted with Gd and Ni.^28,29^ A 0.2 wt % Gd-doped MCM-41-supported Ni catalyst had a GdNi_5_ phase (2–10 nm crystallite), which prevented the agglomeration of Ni and provided oxygen atoms for carbon deposit oxidation.^29^ The addition of just 0.1 wt % Gd over the MCM-41 (or Al_2_O_3_)-supported Ni catalyst caused retention of the size of active metal Ni before and after the reaction ensuring coke resistance and high catalytic performance.^30^ Over silica-supported Ni, Gd/Ni = 0.45 was found to enhance the DRM activity due to enhanced CO_2_ adsorption (formation of surface carbonate species), increased metal–support interaction, and Ni dispersion.^31,32^ The promotional addition of 0.5% Gd_2_O_3_ caused a stronger interaction with Ru (causes smaller Ru particle) over the Zr_0.5_Ce_0.5_O_2_ support and decreased the apparent activation energy of methane conversion.^33^ A 1 wt % Gd-promoted yttria-supported Ni catalyst had easy reducibility, high surface area, high basicity, and strong carbon resistance compared to the nonpromoted one.^34^ Zhang et al. prepared a Gd-promoted alumina-silica-supported Ni catalyst by the one-pot Pechini method and found that 1.2 wt % Gd addition tended to weaken NiAl_2_O_4_ formation, facilitate the reduction of Ni, and enhance the catalytic activity of CH_4_:CO_2_:N_2_ = 35:35:20 gas feed.^35^ ZrO_2_-Gd_2_O_3_ (90:10 mol ratio) solid solutions were sinter reactive.^36^ The Zr^+4^ cation was too small (*r* = 0.084 nm) to support a full eightfold oxygen coordination. Increasing the dopant size like Gd^+3^ led to an increase in the concentration of the vacancies. Among Gd^+3^, Y^+3^, Yb^+3^, and Nd^+3^, doping of Gd^+3^ caused an increase in conductivity.^37^

Overall, Gd was successfully incorporated in various Ni-containing supports like silica, alumina, alumina–silica, yttria, and zirconia and tested for DRM. The oxygen endowing capacity of zirconia, stabilization of zirconia by yttria, and oxide layer enrichment over yttria-zirconia has drawn much interest to use yttria-zirconia as support for DRM. Herein, we have investigated a Gd-promoted yttrium-zirconia-supported Ni catalyst system for hydrogen production through DRM. Again, a simple catalyst preparation procedure is needed to make it handy for less skilled workers in industry. Here, the catalyst is prepared through simple steps like mechanical mixing followed by calcination. The phase distribution, Ni coordination environment and band gap between valence and conduction bands, CO_2_-bonding surface species, and presence of reducible NiO-interacting species are studied by X-ray diffraction, Raman spectroscopy, UV–vis spectroscopy, and infrared spectroscopy, respectively. The retention of Ni active sites during DRM reaction are studied by a H_2_-TPR → CO_2_-TPD → H_2_TPR cyclic experiment over a fresh catalyst. Morphology is depicted by transmission electron microscopy, and last weight loss (%) of the spent catalyst system is investigated by thermogravimetric analysis. By comparison of different activities (like H_2_-yield (*Y*_H_2__), CO yield (*Y*_CO_), CH_4_ conversion (*C*_CH_4__), and *Y*_H_2__/*C*_CH_4__)), the possible DRM reaction mechanism and competitive RWGS reaction are outlined. Finally, a scientific correlation of characterization results and catalytic activity over the Ni-Gd/Y + Zr catalyst system is determined.

## 2 Experimental Section

### 2.1 Materials

Materials used were Ni (NO_3_)_2_^.^6H_2_O (98%; Riedel-de Haen AG, Seelze, Germany), Gd (NO_3_)_2_^.^6H_2_O (99.9%; Ventron, Alfa Produkte), Y_2_O_3_ (Daiichi Kigenso Kagaku Kogyo Co., Ltd., Japan), and ZrO_2_ (Daiichi Kigenso Kagaku Kogyo Co., Ltd., Japan).

### 2.2 Catalyst Preparation

The wet impregnation method was used to prepare the zirconium-supported Ni catalyst, yttrium-zirconium-supported Ni catalyst, and gadolinium-promoted yttrium-zirconium-supported nickel catalyst. Yttrium and zirconium oxides were mixed together mechanically. An aqueous solution of (5 wt %) nickel nitrate precursor and (1.0, 2.0, 3.0, 4.0, 5.0 wt %) gadolinium nitrate using purified water were then added to the support (8 wt % yttrium-zirconium). The prepared mixture was stirred at 80 °C and dried at 120 °C overnight in an oven. Subsequently, the dried product was calcined at 600 °C for 3 h. The zirconium-supported Ni catalyst and gadolinium-promoted yttrium-zirconium-supported nickel catalyst were abbreviated as 5Ni/Zr and 5Ni*x*Gd/Y + Zr (0, 1, 2, 3, 4, 5), respectively. The scanning electron microcopy image and energy-dispersive X-ray (EDX) elemental analysis of the 5Ni4Gd/Y + Zr and 5Ni5Gd/Y + Zr catalysts are shown in Figure S1. SEM images show the salty texture of the catalysts, and EDX analysis shows the presence of all claimed elements in synthesis. Upon increasing Gd loading, the atomic percentage of Gd is also found to increase. The surface area, pore volume, and pore diameter of the Gd-promoted catalysts were found lower than the unpromoted catalysts (Figure S2). No substantial changes in surface parameter is observed upon increasing Gd loading. In the Gd-promoted catalyst system, the surface area, pore volume, and pore diameter were typically in the 26–27 m^2^/g, 14–16 cm^3^/g, and 21.36–23.49 nm-pore-diameter ranges, respectively.

### 2.3 Catalyst Characterization

The catalysts were characterized by X-ray diffraction (XRD), Raman spectroscopy, Fourier-transform infrared spectroscopy (FTIR), ultraviolet–visible spectroscopy (UV–vis), transmission electron microscopy (TEM), H_2_ temperature-programmed reduction (H_2_-TPR), CO_2_ temperature-programmed desorption (CO_2_-TPD), thermogravimetric analysis (TGA), and O_2_ temperature-programmed oxidation (O_2_-TPO). The detailed description of instruments and characterization procedure is given in the Supporting Information.

### 2.4 Catalyst Activity Test

The dry reforming methane catalytic activity test started with a 1 g catalyst put in a stainless-steel vertical fixed tubular reactor (9.1 mm i.d. and 30 cm long) (PID Eng & Tech Micro Activity) using a ball of glass wool. The reaction was carried out under atmospheric pressure. A K-type stainless sheathed thermocouple was used to maintain the temperature of the reaction. The catalysts were activated with reductive treatment under the flow of hydrogen (20 mL/min) for 60 min at 600 °C. The mixture of feed gas was CH_4_/CO_2_/N_2_ at 30, 30, 10 mL/min (with a space velocity of 42,000 mL/h·g_cat_) passed through the catalyst at 800 °C. DRM reaction was progressed, and the effluent was analyzed by an online GC-2014 Shimadzu (Molecular Sieve 5A and Porapak Q columns) equipped with a thermal conductivity detector under Ar carrier gas. The H_2_ yield %, CO yield %, CH_4_ conversion, CO_2_ conversion, and carbon formation rate were determined by the following expressions.
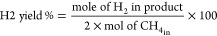

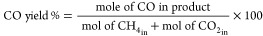

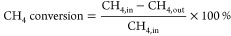

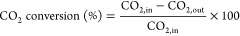

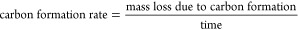


## 3 Results

### 3.1 Catalytic Activity Results

**Figure 1:**
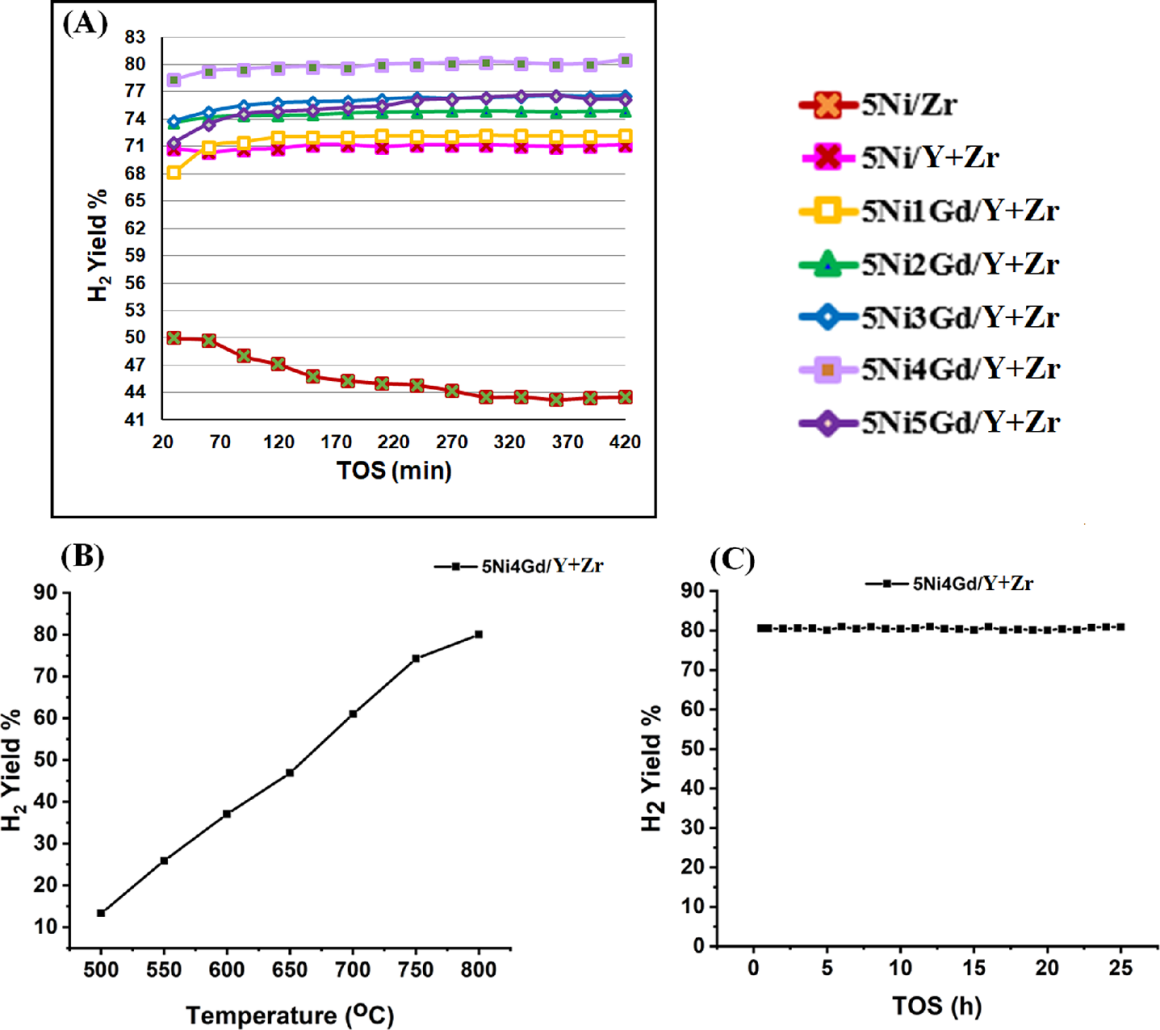
(A) H_2_ yield over Ni/Zr and Ni*x*Gd/Y + Zr (*x* = 0–5) catalyst. (B) H_2_ yield over 5Ni4Gd/Y + Zr at different reaction temperatures. (C) Long time on stream (TOS) study over 5Ni4Gd/Y + Zr.

The catalytic activities of the promoted and nonpromoted catalysts at 800 °C for 420 min are shown in Figure 1A. The zirconia-supported Ni catalyst (5Ni/Zr) shows the lowest H_2_ yield (50%), which drops to 43% at the end of 420 min. When yttrium is added to the zirconia support, the catalytic activity jumps to 70% H_2_ yield, which remains constant for up to 420 min. This means that 5Ni/Y + Zr got higher and stable catalytic activity than the 5Ni/Zr catalyst. 1 wt % addition of the Gd promoter to the yttrium-zirconia-supported Ni catalyst (5Ni1Gd/Y + Zr) results in further progress of catalytic activity to 71%. Incorporating more wt % of the Gd promoter up to 4 wt % into the yttrium-zirconia-supported Ni catalyst (5Ni*x*Gd/Y + Zr; *x* = 2,3,4), the H_2_ yield is increased progressively. 5Ni4Ga/Y + Zr shows the highest H_2_ yield (78%) up to 420 min. More than 4 wt % Gd incorporation into the yttrium-zirconia-supported Ni catalyst results in a drop in catalytic activity. 5Ni4Gd/Y + Zr is found best in mean of H_2_ yield toward DRM, and so this catalyst system is further investigated at different reaction temperatures for DRM reaction. It is found that the H_2_ yield increases monotonically as the reaction temperature is increased from 500 up to 800 °C over the 5Ni4Gd/Y + Zr catalyst (Figure 1B). The 5Ni4Gd/Y + Zr catalyst is further tested for a long time on stream, and 80% H_2_ yield is obtained at 800 °C constantly for 24 h of time on stream (Figure 1C).

As per the DRM reaction, the H_2_ yield and CO yield should be equal. However, it is observed that the CO yield always remains higher than the H_2_ and CO_2_ yields over each catalyst system (Figure S3). It indicates that along with DRM reaction, another H_2_-consuming reaction also takes place over the catalyst system. A reverse water gas–shift reaction (RWGS) (CO_2_ + H_2_ → CO + H_2_O) is a thermodynamic feasible reaction, which happens in the same temperature range as that of DRM.^38^ It is noticeable that during the 420 min time on stream, the CO yield is 30–40% higher than the H_2_ yield over the 5Ni/Zr catalyst, 7–10% higher than the H_2_ yield over the 5Ni/Y + Zr catalyst, and 4–5% higher than the H_2_ yield over the 5Ni*x*Gd/Y + Zr (*x* = 4,5) catalyst system. It indicates the successive suppression of the RWGS reaction over the gadolinium-promoted yttria-zirconia-supported Ni catalyst system.

The H_2_ yield, CH_4_ conversion, CO yield, and CO_2_ conversion over the 5Ni/Zr and 5Ni*x*Gd/Y + Zr (*x* = 0, 1, 2, 3, 4, 5) catalyst system at the end of 60 min are shown in Figure S3C. If it is proposed that CH_4_ is dissociated into C + 2H_2_, then the stoichiometric *Y*_H_2__/*C*_CH_4__ ratio for CH_4_ decomposition should be 2. *Y*_H_2__/*C*_CH_4__ < 2 may be indicative of the involvement of H_2_ in the RWGS reaction potentially. It may also indicate the formation of CH_*y*_ and expulsion of H_2_ gas after decomposition of CH_4_.^39,40^ In our case, the H_2_ yield (*Y*_H_2__) is found always less than CH_4_ conversion (*C*_CH_4__) and the CO yield remains less than CO_2_ conversion. Simply, the *Y*_H_2__/*C*_CH_4__ ratio remains between 0.95 and 0.99 over the 5Ni/Zr and 5Ni*x*Gd/Y + Zr (*x* = 0, 1, 2, 3, 4, 5) catalyst system. Earlier, we have found that the RWGS reaction is suppressed potentially over the 5Ni4Gd/Y + Zr and 5Ni5Gd/Y + Zr catalyst. However, over these catalysts, also the *Y*_H_2__/*C*_CH_4__ ratio remains between 0.95 and 0.97. It indicates that over the 5Ni*x*Gd/Y + Zr (*x* = 0, 1, 2, 3, 4, 5) catalyst system, CH_4_ is decomposed into CH_y_ and  H_2_ (not into C and H_2_).

### 3.2 Catalyst Characterization Results

#### 3.2.1 XRD Study

**Figure 2:**
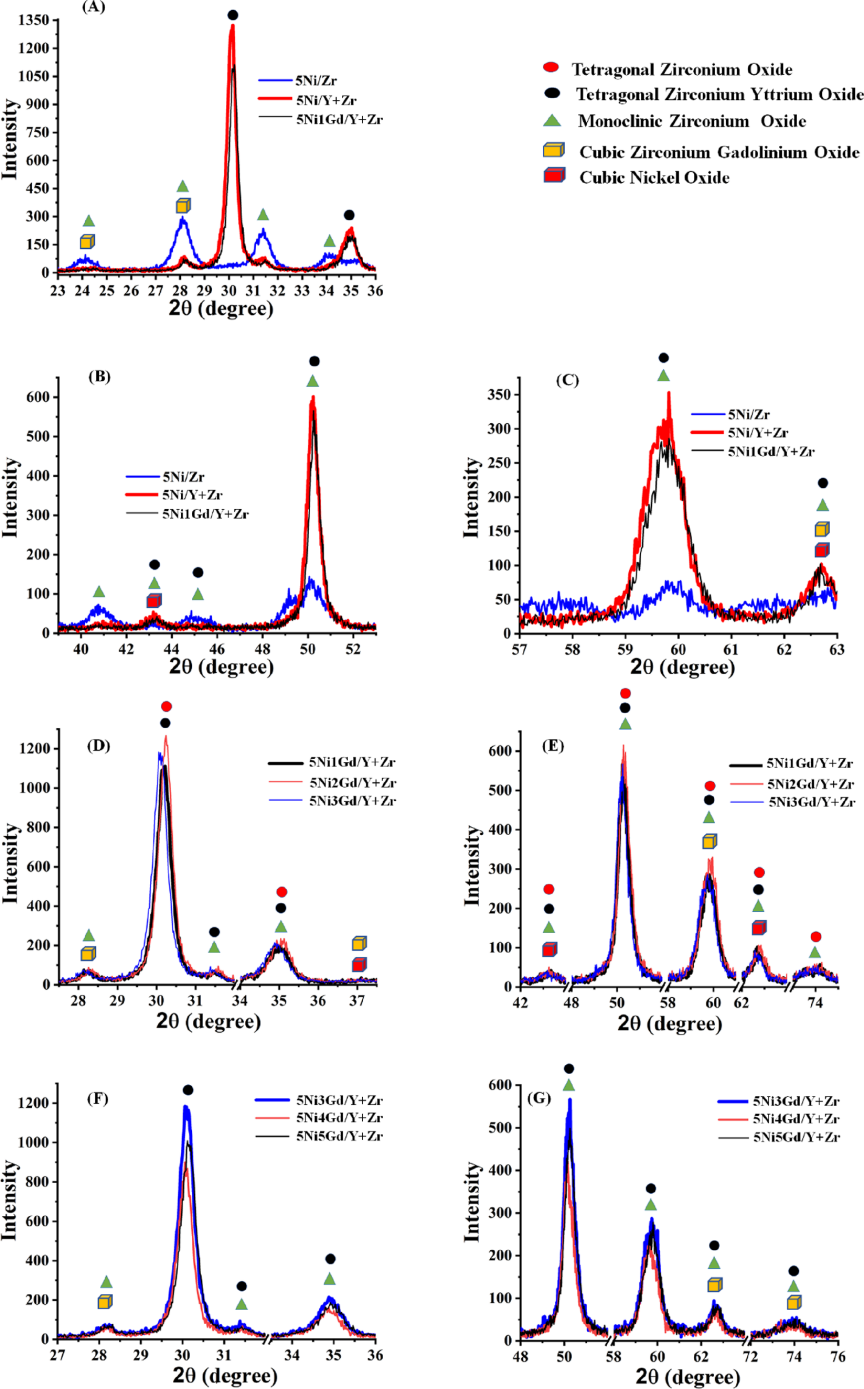
(A–C) XRD profiles of fresh 5Ni/Zr and 5Ni1Gd/Y + Zr. (D,E) XRD profiles of 5Ni*x*Gd/Y + Zr (*x* = 1, 2, 3) catalysts. (F,G) XRD profile of 5Ni*x*Gd/Y + Zr (*x* = 3, 4, 5) catalysts.

XRD of fresh promoted and unpromoted catalysts are shown in Figure 2. The zirconia-supported nickel catalyst shows a monoclinic zirconia phase (at 2θ = 24.25, 28.16, 31.42, 34.0, 35.32, 38.5, 40.8, 44.7, 49.13, 50.21, 54.0, 55.40, 59.88, 62.77, 65.7, 71.04, 75.2°; JCPDS card reference number 00-007-0343) and cubic NiO (at 2θ =, 37.40, 43.17, 62.73°; JCPDS card reference number 00-004-0835). The crystallite size of NiO was found at 7.6 nm (Figure 2A–C). However, in the yttria-zirconia-supported Ni catalyst, such peaks are suppressed markedly; tetragonal zirconium yttrium oxide peaks (at 2θ = 30.14, 35.00, 43.2, 50.13, 60.0, 62.73, 73.83°; JCPDS card reference number 01-082-1241) are prominent. The size of cubic NiO crystallite is about ∼13 nm over 5Ni/Y + Zr. This means tetragonal yttria stabilize the tetragonal phase of zirconia by forming a mixed tetragonal zirconia yttrium oxide phase. Simply, doping of zirconia with a lower-valence ion Y^+3^ (radius of Y^+3^ = 1.06 Å) was found to stabilize the tetragonal phase of zirconia above 400 °C.^41,42^ The valence of yttrium is smaller than that of zirconium. Lower-valence Y^+3^ serves as a substitute for the Zr^+4^ ion; the Y^+3^ ion is incorporated into the zirconia crystallite structure and stabilizes the tetragonal phase of zirconia. Furthermore, tetragonal phases of yttria and tetragonal phases of zirconia turn into the formation of a mixed tetragonal zirconia-yttrium oxide phase. The cubic zirconium gadolinium oxide phases (at 2θ =24.34, 28.22, 37.19, 62.66°; JCPDS card reference number 01-080-0469) are also noticed over the 5Ni1Gd/Y + Zr catalyst, but these peaks are generally merged with the zirconia phase peaks (Figure 2A–C). It seems that due to the engagement of the ZrO_2_ phase with Gd, the peak intensity of the ZrO_2_-related phases decreased upon Gd introduction. On further 3 wt % Gd addition, the peak intensity not only decreased but also shifted toward the lower brag angle relatively, which indicated the lattice expansion on 3 wt % Gd loading (Figure 2D,E). On gadolinium addition at 3–5 wt %, the peak intensity decreased continuously (Figure 2F,G). It indicates that Gd addition decreases the crystallinity and increases the amorphousity. Interestingly, on 4 wt % Gd promotional addition, the NiO crystallite size is a minimum of 7.5 nm (Table 1). In the spent catalyst system, all crystalline phases decrease and no crystalline carbon phases are observed (Figure S4). Over the spent 5Ni*x*Gd/Y + Zr (*x* = 0, 1, 2, 3, 5) catalyst system, the metallic Ni phase (at Bragg angle 44.50°; JCPDS card reference number 00-004-0850) is evident. Over the spent 5Ni1Gd/Y + Zr catalyst, a 15.6 nm metallic Ni phase is noticed whereas the Ni size is optimized to 7.6 nm over the spent 5Ni*x*Gd/Y + Zr (*x* = 2–3) catalyst (Table 1). Interestingly, over the spent 5Ni4Gd/Y + Zr catalyst, no diffraction peaks for the metallic Ni phase is observed. It indicates the dispersion of metallic Ni over the spent catalyst system. The role of Gd in size optimization of Ni species was noticeable in the fresh as well as spent catalyst system.

**Table 1:** Crystalline Size of NiO in Fresh Catalysts and Ni in Spent Catalysts over Different Catalyst Systems

catalyst name	NiO crystallite size in fresh catalyst	Ni crystallite size in spent catalyst
5Ni/Zr	7.6	15.2
5Ni/YZr	12.9	15.2
5Ni 1Gd/Y + Zr	10.1	15.2
5Ni 2Gd/Y + Zr	11.3	7.6
5Ni 3Gd/Y + Zr	15.1	7.6
5Ni 4Gd/Y + Zr	7.5	
5Ni 5Gd/Y + Zr	9.0	7.6

#### 3.2.2 Raman and Infrared Spectroscopy

**Figure 3:**
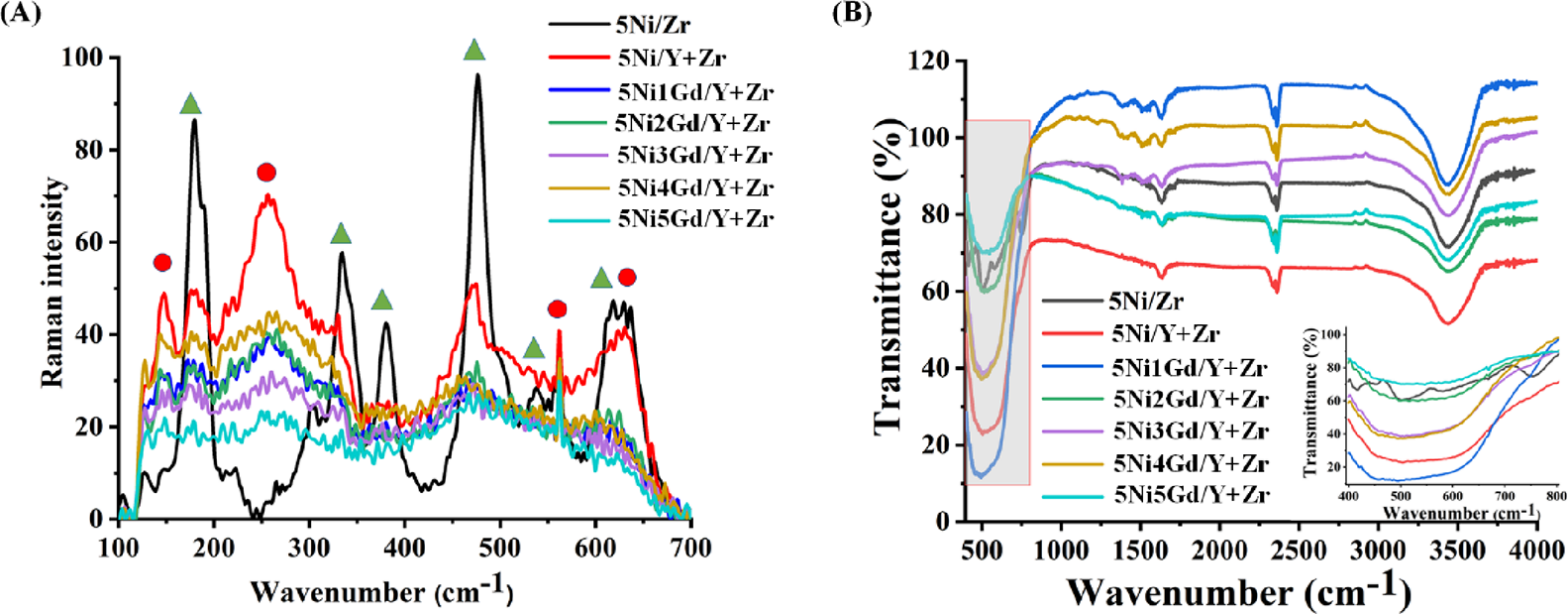
(A) RAMAN spectra of the 5Ni/Zr and 5Ni*x*Gd/Y + Zr (*x* = 0, 1, 2, 3, 4, 5) catalysts. (B) Infrared spectra of the 5Ni/Zr and 5Ni*x*Gd/Y + Zr (*x* = 0, 1, 2, 3, 4, 5) catalysts; inset: infrared spectra of the 5Ni/Zr and 5Ni*x*Gd/Y + Zr (*x* = 0, 1, 2, 3, 4, 5) catalysts in the 400 to 800 cm^–1^ wavenumber range.

The XRD results are also supported by Raman spectra. The Raman profile of the promoted and unpromoted catalysts is shown in Figure 3A. The peaks at 179, 334, 380, 476, and 610 cm^–1^ characterize the monoclinic zirconia where the 476 cm^–1^ peak is larger than that of 610 cm^–1^.^43^ The peak at 630 cm^–1^ characterizes tetragonal zirconia for the zirconia-supported nickel catalyst. The yttria-zirconia-supported Ni catalyst has a low-intensity monoclinic zirconia peak but an emerging tetragonal zirconia peak at 146, 260, and 625 cm^–1^.^44^ In the 1 wt % Gd (over 5Ni/Y + Zr catalyst), the intensities of all major peaks decrease (Figure 3A). These observations are in line with the XRD results (discussed above). Infrared spectra of fresh promoted and unpromoted catalysts are shown in Figure 3B. Infrared spectra of all catalyst samples show vibration peaks of O–H at 1630 and 3444 cm^–1^. ZrO_2_-supported Ni had Zr–O vibration peaks at 498 and 750 cm^–1^.^11^ The free NiO in a cubic lattice was reported at 433 cm^–1^ wavenumbers, whereas in the ZrO_2_-supported Ni catalyst, a Ni–O– vibration peak at a lower wavenumber (420 cm^–1^) was observed.^45^ It indicates the deformation of cubic NiO species and formation of “NiO-interacted support” species, which result in the weakening of the Ni–O bond and vibration of Ni–O at a lower wavenumber than in free Ni–O in a cubic lattice. However, in the yttria-zirconia-supported Ni catalyst, all Ni–O and Zr–O vibration peaks disappear. It indicates that the presence of yttria brought a major change in bonding patterns. IR of the 5Ni*x*Gd/Y + Zr (*x* = 0,1,2,3,4,5) catalyst system shows the presence of CO_2_ as monodentate carbonate species at 1388 and 1516 cm^–1^^46^ as well as physically adsorbed CO_2_ at 2343–2352 cm^–1^.^11^

#### 3.2.3 Ultraviolet–Visible Spectroscopy

**Figure 4:**
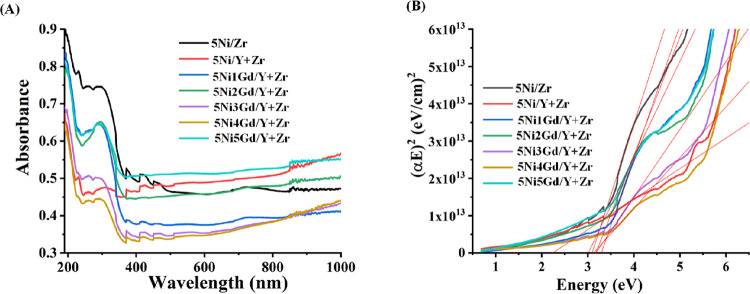
(A) Ultraviolet–visible (UV–vis) spectroscopy of 5Ni/Zr and 5Ni*x*Gd/Y + Zr (*x* = 0, 1, 3, 4, 5, 6) catalysts. (B) Band gap of 5Ni/Zr and 5Ni*x*Gd/Y + Zr (*x* = 0, 1, 3, 4, 5, 6) catalysts.

The UV spectra and band gap of the 5Ni*x*Gd/Y + Zr (*x* = 0, 1,2,3,4,5) catalyst system are shown in Figure 4. The zirconia-supported nickel fresh catalyst shows a peak at 230 nm for charge transfer of O^–2^ to Zr^+4^, “258 and 286 nm” for charge transfer of O^–2^ to Ni^+2^ in the octahedral symmetry,^24,47^ peaks at 371, 410, and 451 nm for the d–d transition from ^3^A_2g_ to ^3^T_1g_(P)^48^ of Ni^+2^ in the octahedral environment, and peak at 717 nm for the d–d transition from ^3^A_2g_ to ^3^T_1g_(F) of Ni^+2^ in the octahedral environment.^12^ Altogether, UV–vis spectra confirm the octahedral coordination of Ni in the zirconia-supported catalyst. It has a 3.11 eV band gap. The yttria-zirconia-supported catalyst had the same type of peak pattern and the least band gap (2.23 eV) with respect to the rest of the catalysts. It indicates that yttria incorporation does not change the coordination environment but the charge transfer of O^–2^ from the valence band to the conduction band becomes easier. Upon Gd loading, a peak at 230 nm (for charge transfer of O^–2^ to Zr^+4^) was diffused, which indicates that Gd loading inhibits such transition. The UV–vis spectra of 2 wt % Gd and 5 wt % Gd are noticed by the absence of the d–d transition peaks. Upon 4 wt % Gd loading, the lowest band gap (or similar to the nonpromoted catalyst; 5Ni/Y + Zr) is noticed (Table S1).

#### 3.2.4 Temperature-Programmed Study

**Figure 5:**
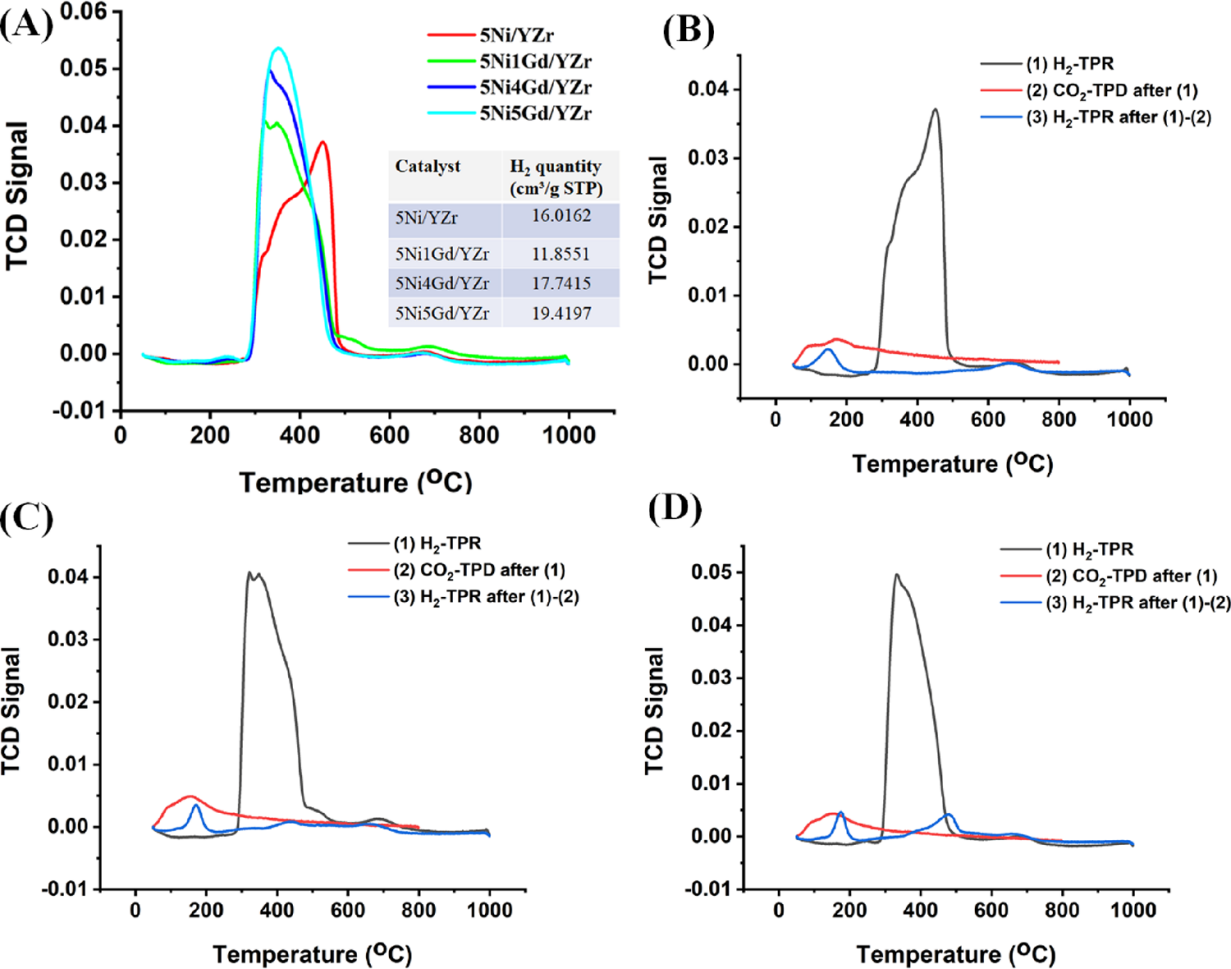
(A) H_2_-TPR of 5Ni*x*Gd/Y + Zr (*x* = 0, 1, 4, 5). (B) H_2_-TPR → CO_2_-TPD → H_2_TPR cyclic experiment of 5Ni/Y + Zr. (C) H_2_-TPR → CO_2_-TPD → H_2_TPR cyclic experiment of 5Ni1Gd/Y + Zr. (D) H_2_-TPR → CO_2_-TPD → H_2_TPR cyclic experiment of 5Ni4Gd/Y + Zr.

In an XRD study of the fresh catalyst system, a cubic NiO phase is found, which is reducible (into metallic Ni) under H_2_ stream. The amount of reducible NiO over different temperatures reflects the extent of interaction of such NiO species over the support. Now, to understand the type of reducible species over the fresh catalyst system, H_2_-TPR is discussed in more detail. Al-Fatesh et al. showed that H_2_-reduction peaks before 200 °C are for reducible free NiO species over the zirconia-supported Ni catalyst.^49^ In our case, there are no reduction peaks before 200 °C in the 5Ni*x*Gd/Y + Zr (*x* = 0–5) catalyst system (Figure 5A and Figure S5). It indicates that upon introducing yttria or both yttria and gadolinium, all NiO species interact with the support. The H_2_-TPR peak pattern of the 5Ni*x*Gd/Y + Zr (*x* = 0, 1, 4, 5) catalyst system is composed of peaks about 330 °C for reducible “NiO species weakly interacting with the support,” shoulder peaks about 350 °C for reducible “NiO species moderately interacting with the support”, and high-temperature peaks about 465 °C for reducible “NiO species strongly interacting with the support”.^21^ It is interesting to note that on introducing 1 wt % Gd, peak patterns of lower and intermediate temperatures increase at the expense of a high-temperature peak. This means that the amount of weak and moderately reducible NiO-interacted species is growing over the surface upon Gd introduction whereas hardly reducible NiO-interacted species are decreasing. On increasing loading of up to 5 wt % Gd, H_2_ consumption is increasing in lower and intermediate temperatures continuously. It indicates that the amount of reducible NiO species keeps increasing on Gd increase of loading.

Before the DRM reaction, the catalyst is pre-treated under H_2_ and NiO is reduced into Ni, which is a catalytic active site. Furthermore, CH_4_, CO_2_, and N_2_ gas feed are passed over the catalyst at a reaction temperature of 800 °C. CO_2_ is an oxidizing gas, which can oxidize the carbon deposit as well as metallic Ni. Oxidation of Ni to NiO may turn the catalyst inactive because of depletion of the active site. It is important to note how our catalyst system behaves in front of CO_2_ stream. To understand this, the 5Ni*x*Gd/Y + Zr (*x* = 0, 2, 4, 5) catalyst is sequentially treated with H_2_-TPR then CO_2_-TPD and then H_2_-TPR (known as a H_2_-TPR → CO_2_-TPD → H_2_TPR cyclic experiment, depicted in Figure 5B–D and Figure S6). It is found that fresh catalysts consumed H_2_ prominently during reduction of the H_2_-TPR and catalysts. Thereafter, CO_2_-TPD of the reduced catalysts shows very little desorption of CO_2_ by the reduced catalysts. Finally, treating the catalyst again with H_2_-TPR in sequence (H_2_-TPR → CO_2_-TPD → H_2_TPR), there is very little consumption of H_2_. It indicates that most of Ni is not oxidized and remains present under CO_2_ stream.

**Figure 6:**
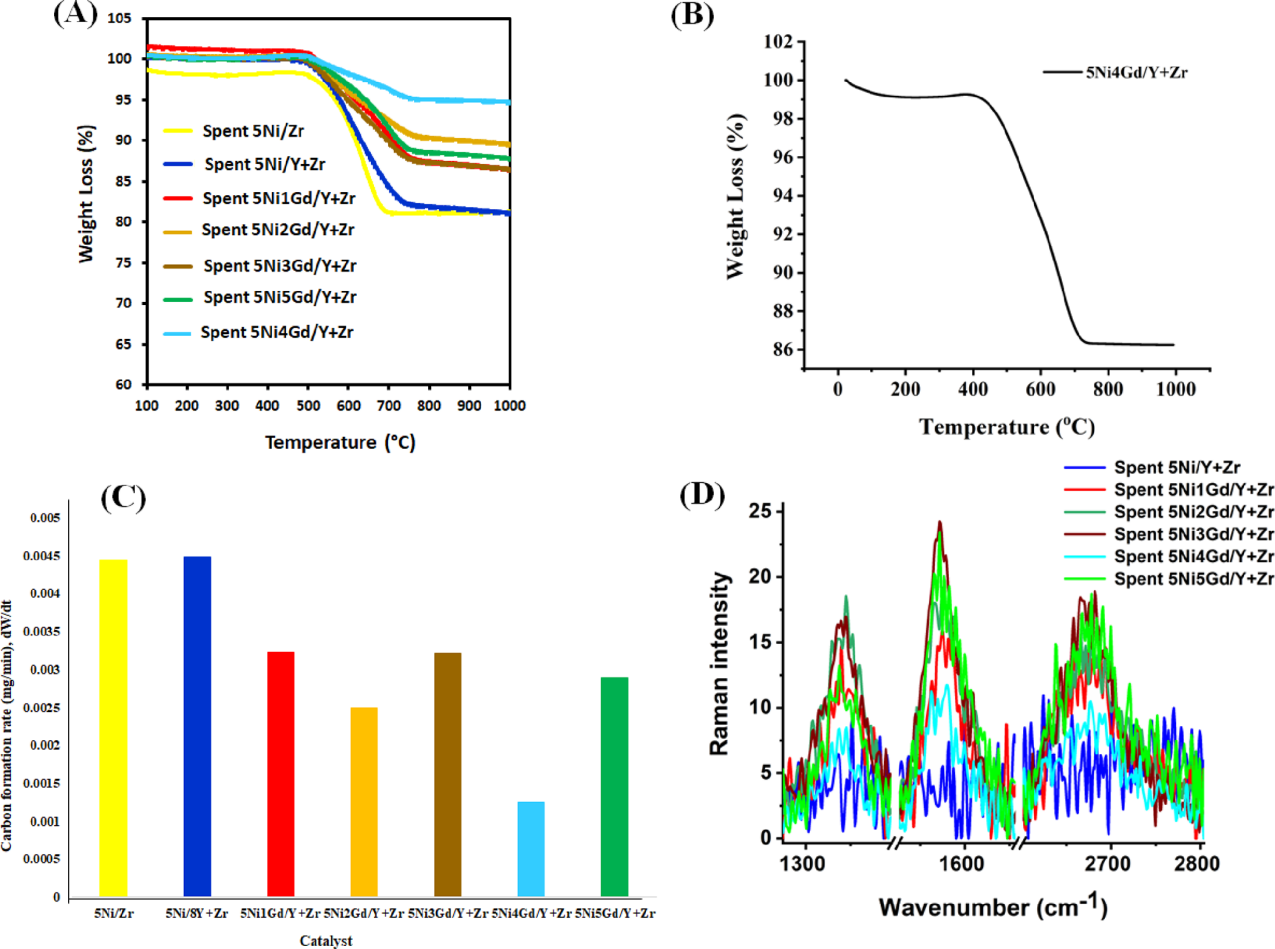
(A) Thermogravimetric analysis (TGA) of the 5Ni/Zr and 5Ni*x*Gd/Y + Zr (*x* = 0, 1, 3, 4, 5) catalysts. (B) Thermogravimetry analysis of spent 5Ni4Gd/Y + Zr (collected after 24 h DRM reaction). (C) Carbon formation rate over different catalysts. (D) Thermogravimetric analysis (TGA) of the spent 5Ni*x*Gd/Y + Zr (*x* = 0, 2, 1, 3, 4, 5) catalysts.

TGA profiles of promoted and unpromoted spent catalysts after 7 h of DRM reaction are shown in Figure 6A. The unpromoted catalysts show a 19% weight loss. 1, 2, 3, 4, and 5 wt % Gd-promoted catalysts show 14, 11, 14, 6, and 13% weight losses. This result shows that 4 wt % Gd-promoted catalyst has the lowest carbon deposition over the surface. Even after a 25 h reaction, the spent 5Ni4Gd/Y + Zr catalyst only showed 12.5% weight loss (Figure 6B). The carbon formation rate over different catalysts is shown in Figure 6C. The 5Ni4Gd/Y + Zr catalyst shows a minimum carbon formation rate (0.001255 mg/min) than other catalysts. To understand the type of carbon species present over the spent 5Ni4Gd/Y + Zr catalyst (after 24 h of DRM reaction), O_2_ temperature-programmed oxidation is carried out (Figure S7). The catalyst showed a single peak in the region of 400–700 °C (peak maxima at 615 °C) for β-carbon species.^12,22^ The Raman spectra of the spent 5Ni4Gd/Y + Zr catalyst showed a “defect carbon band” (*I*_D_) at 1336 cm^–1^, a “graphite band” (*I*_G_) band at 1573 cm^–1^, and a 2D band at 2673 cm^–1 52,59,60^ (Figure S8). Among all the Gd-promoted catalysts, the 5Ni4Gd/Y + Zr catalyst had a minimum peak intensity for the *I*_D_ band, *I*_G_ band, and 2D band (Figure 6D).

#### 3.2.5 Transmission Electron Microscopy

**Figure 7:**
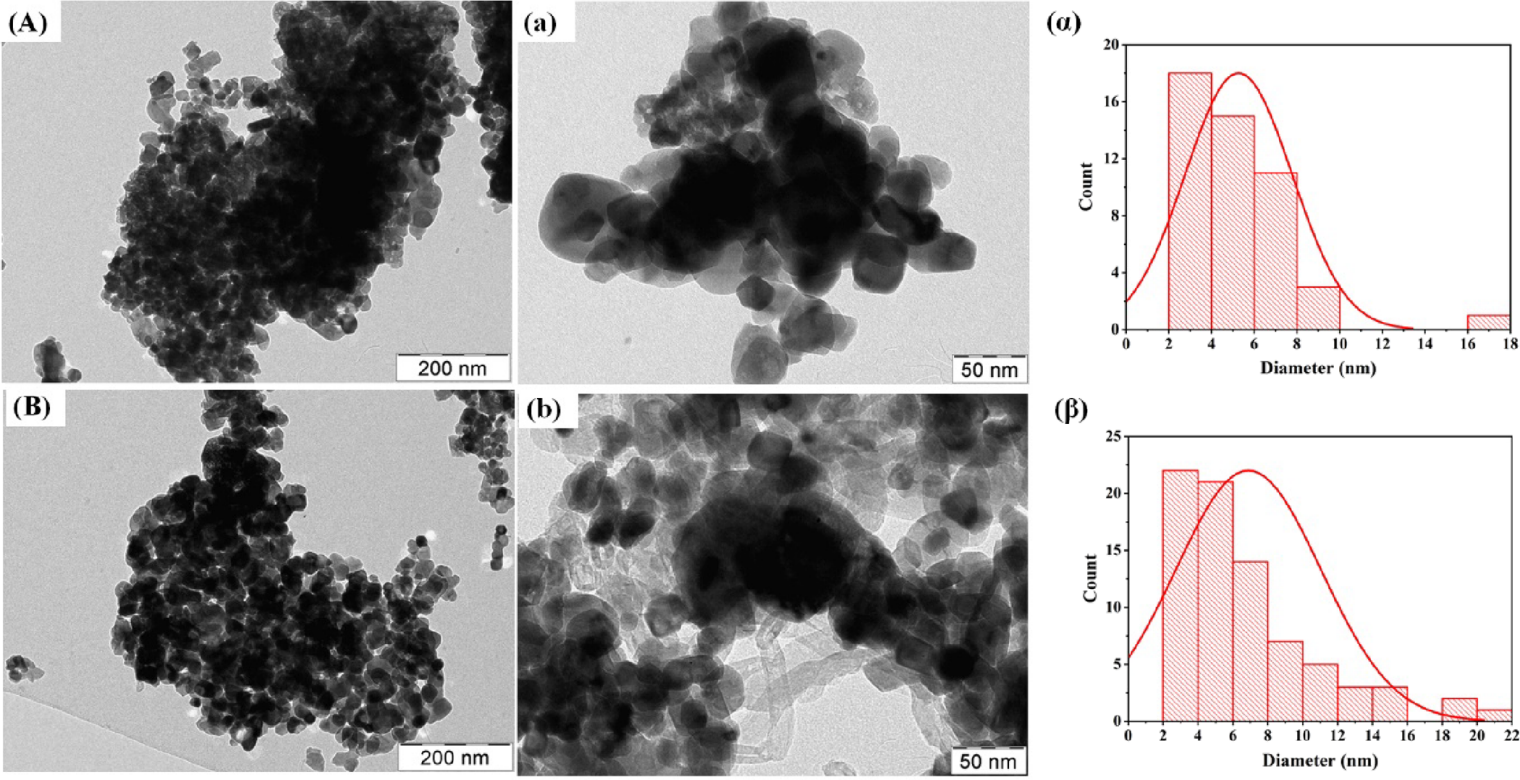
(A, a) TEM images of fresh 5Ni4Gd/Y + Zr catalysts. (α) Particle size distribution of fresh 5Ni4Gd/Y + Zr catalysts. (B, b) TEM images of spent 5Ni4Gd/Y + Zr catalysts. (β) Particle size distribution of spent 5Ni4Gd/Y + Zr catalysts.

TEM images of fresh and spent samples of the 4 wt % gadolinium-promoted yttrium-zirconia-supported nickel catalyst are shown in Figure 7. The fresh catalyst shows a spherical shape with a particle size of 5.21 nm, while the spent catalyst shows a carbon nanotube with encapsulated carbon particle size 6.79 nm. After catalytic reaction, the particle size is increased.

## 4 Discussion

The ZrO_2_ support is known for the presence of dual sites (acid–base sites), which can withstand and hold the active metal species Ni against high-temperature DRM conditions.^51^ From X-ray diffractograms and Raman spectra, mostly unstable monoclinic zirconia phases and cubic NiO crystallites of crystallite size 7.6 nm are confirmed over the Ni/ZrO_2_ catalyst. The UV profile of the catalyst confirms the octahedral sites of Ni^+2^ over the ZrO_2_ support and the 3.11 eV band gap between the valence and conduction bands. Infrared spectra confirm the presence of CO_2_-interacting surface species. The “H_2_-TPR → CO_2_-TPD → H_2_TPR” cyclic experiment over the fresh catalyst system shows that most of the Ni (catalytic active sites) remained present even under oxidizing CO_2_ stream. However, the catalytic activity toward DRM was found inferior (43% H_2_ yield at 420 min). Al-Fatesh et al. showed that zirconia-supported Ni catalysts had reducible free NiO species (less interacting NiO species) over the catalyst surface.^49^ Overall, the inferior activity of Ni/Zr toward DRM can be claimed to show unstable monoclinic zirconia phases, less-interacting NiO species, and a huge carbon deposit (∼19% weight loss in TGA). The CO yield over the 5Ni/Zr catalyst remains 30–40% higher than the H_2_ yield during the 420 min time on stream. It indicates that after DRM, the major competitive reaction is RWGS over the 5Ni/Zr catalyst.

Yttria addition along with zirconia support has brought major physiochemical changes in the catalytic property of the surface. XRD and Raman confirm the presence of a stable tetragonal zirconia phase through the formation of the tetragonal zirconia-yttrium oxide (mixed oxide) phase. The band gap between the valence band and the conduction band decreased sharply after yttrium incorporation (2.23 eV). 5Ni/Y + Zr had reducible “NiO surface-interacted species” in which strongly interacted NiO species are a majority. These favorable surface properties over the 5Ni/Y + Zr catalyst push the H_2_ yield up to 70%, and the H_2_ yield remains constant till 7 h over a tested time. However, carbon deposition over the catalyst surface is not improved enough. The weight loss (%) remains 14% over the spent 5Ni/Y + Zr catalyst. Interestingly, despite carbon deposition, catalysts maintain high catalytic activity constantly. Possibly the rate of carbon formation may properly match the rate of carbon diffusion away from the catalytic active center, which affects the catalytic activity to a less extent.^52^ The CO yield remains 7–10% higher than the H_2_ yield. It indicates that the RWGS reaction is suppressed greatly over the 5Ni/YZr catalyst than the 5Ni/Zr catalyst.

Definitely from here, the carbon deposition problem should be overcome to achieve higher activity up to the industrial mark. Upon 1 wt % Gd promotional addition, a cubic zirconium-gadolinium oxide phase is built. This means now that zirconia is stabilized by both tetragonal zirconium-yttrium oxide and cubic zirconium-gadolinium oxide phases. The crystallinity of the catalyst is decreased, along with the amount of weakly and moderately interacted NiO species growing on the surface at the expense of strongly interacted NiO species. On increasing Gd incorporation up to 4 wt %, the crystalline size of NiO is decreased to the lowest value of 7.5 nm, the crystallinity of catalyst is decreased continuously, the band gap between the conduction and valence bands is decreased to the lowest magnitude of 2.23 eV (similar to 5Ni/Y + Zr), and the weakly and moderately interacted reducible NiO species are mounted over the catalyst surface. It was reported that controlling the Ni particle ensemble size <9 nm restricts the thermal sintering of Ni particles, enhances the metal–support interaction, and alleviates the carbon deposition.^53^ Here also, the 5Ni4Gd/Y + Zr catalyst has a minimum size of Ni species compared to another Gd-promoted catalyst, and so the minimum coke deposit is expected over it during the DRM reaction. Overall, the 5Ni4Gd/Y + Zr catalyst showed about 78% H_2_ yield constantly up to 420 min with minimum coke deposit (6% weight loss in TGA). The CO yield remains just 4–5% higher than the H_2_ yield over the 5Ni4Gd/Y + Zr catalyst. It indicates that RWGS is greatly retarded over 5Ni4Gd/Y + Zr. The particle size of 5Ni4Gd/Y + Zr is grown from 5.21 to 6.79 nm after the DRM reaction after 7 h. The 5Ni4Gd/Y + Zr catalyst is tested for longer TOS (24 h) where it again shows a constant 80% H_2_ yield with 12.5 wt % mass loss. The deposited carbon above the catalyst surface is β-carbon species having a ratio of both defect carbon and graphite carbon. It is noticeable that upon 5 wt % Gd incorporation, the total number of reducible NiO species increases but the NiO crystallite size and band gap between the valence band and conduction band increase. It results into a relative drop of catalytic activity compared to the 5Ni4Gd/Y + Zr catalyst.

**Figure 8:**
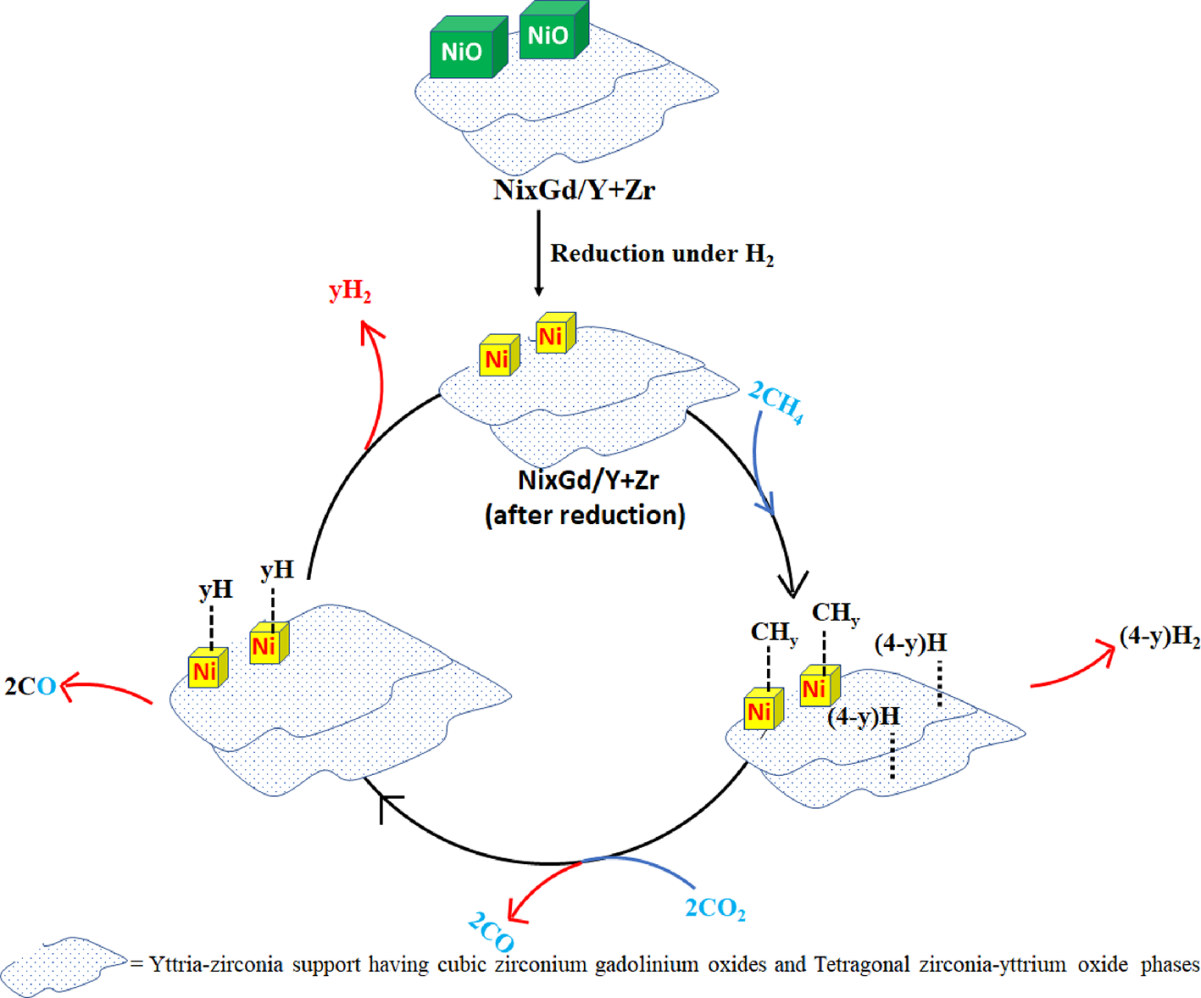
The proposed mechanism of hydrogen production from dry reforming of methane over 5Ni*x*Gd/Y + Zr (*x* = 0–5).

On the basis of characterization results and prior pioneer work in this field, the mechanism of hydrogen production from dry reforming of methane is proposed over the 4 wt % Gd-promoted yttria-zirconia-supported Ni catalyst (Figure 8).^54^ Initially reducible cubic NiO is stabilized over the yttria-zirconia support having stable tetragonal zirconia-yttria oxide and cubic zirconium-gadolinium oxide phases. Upon pre-treatment under H_2_, catalytic active sites or metallic Ni is formed over this support. Furthermore, CH_4_, CO_2_, and N_2_ gas feed are passed over the catalyst at reaction temperature 800 °C. The 5Ni4Gd/Y + Zr catalyst shows great suppression for the RWGS reaction. A low *Y*_H_2__/*C*_CH_4__ ∼0.97 over the 5Ni4Gd/Y + Zr catalyst also confirms the decomposition of CH_4_ into CH_*y*_ and (4-*y*)H over metallic Ni. Furthermore, (4-*x*)H is adsorbed at a nearby surface and is finally desorbed as H_2_. CO_2_ is interacted and dissociated over the surface into “CO” and “O”. Now, “O” is available for oxidation of CH_*x*_ and it may oxidize the catalytic active site metallic Ni. However, over the 5Ni*x*Gd/Y + Zr (*x* = 0–5) catalyst system, metallic Ni is mostly retained even in the presence of oxidizing gas CO_2_ (as shown in the H_2_-TPR → CO_2_-TPD → H_2_TPR cyclic experiment). Last, CH_*x*_ is oxidized into CO and *x*H. Later, *x*H is desorbed as H_2_.

Table 2 shows the comparable catalytic activity of the 5Ni4Gd/Y + Zr catalyst system over other Ni-based stabilized-zirconia catalyst systems. Ni dispersed over different supports like tungsten-zirconia, phosphate-zirconia, lanthana-zirconia, and yttria-zirconia catalyst system was found promising for DRM. At 5 wt % Ni loading, the 8 wt % yttria–92 wt % zirconia support outperformed in the mean of the H_2_ yield (∼71%) than the rest. Furthermore, promotional addition of promotors like ceria, chromium, barium, holmium, and gadolinium over stabilized zirconia boosted the H_2_ yield beyond 80%. The current 5Ni4Gd/YZ catalyst maintained 80% H_2_ yield continuously up to the 24 h TOS.

**Table 2:** Comparable Catalytic Activity of the 5Ni4Gd/Y + Zr Catalyst System over Other Ni-Based Stabilized Zirconia Catalyst Systems^a^

					feed ratio							
sr. no.	catalyst name	MP	wt % Ni	CW (mg)	CH_4_	CO_2_	CG	GHSV L/(g_cat·_h)	TOS (h)	*T* (°C)	*Y*_H_2__ (%)	ref
1	5Ni/WZr	I	5	100	3	3	1	42	7	700	43	(22)
2	10Ni/PZr	I	10	150	3	3	1	28	∼7.5	800	74	(24)
3	5Ni/LaZr	I	5	100	3	3	1	42	8	700	58	(21)
4	5Ni/YZr	I	5	100	3	3	1	42	7	700	71	(50)
5	5Ni2.5Ce/WZr	I	5	100	3	3	1	42	7	700	78	(22)
6	10Ni3Ce/PZr	I	10	150	3	3	1	28	∼7.5	800	97	(24)
7	5Ni1Ga/LaZr	I	5	100	3	3	1	42	8	700	73	(21)
8	5Ni1Ca/LaZr	I	5	100	3	3	1	42	8	700	72	(21)
9	5Ni1Gd/LaZr	I	5	100	3	3	1	42	8	700	80	(21)
10	5Ni1Cr/LaZr	I	5	100	3	3	1	42	8	700	81	(21)
11	5Ni2.5Ce/LaZr	I	5	100	3	3	1	42	∼7	700	87	(11)
12	5Ni2Ce/YZr	I	5	100	3	3	1	28	7	800	80	(55)
13	5Ni3Sr/YZr	I	5	100	3	3	1	42	7	700	62	(56)
14	5Ni4Ba/YZr	I	5	100	3	3	1	42	7	800	80	(57)
15	5Ni4Ho/YZr	I	5	100	3	3	1	42	7	700	84	(50)
16	5Ni4Gd/Y + Zr	I	5	100	3	3	1	42	25	800	80	this study

a MP = method of catalyst preparation, I = impregnation, Wt = weight, CW = catalyst weight, CG = carrier gas, GHSV = gas hour space velocity, TOS = time on stream, *T* = temperature, *Y*_H_2__ = hydrogen yield, ref = reference.

## 5 Conclusions

Over the 5Ni*x*Gd/Y + Zr (*x* = 0–5) system, most of the Ni (catalytic active sites) remained present even under oxidizing CO_2_ stream. The inferior catalytic activity (43% H_2_ yield) of the zirconia-supported Ni catalyst is due to an unstable monoclinic zirconia phase and huge carbon deposition. The use of yttria along with zirconia support stabilizes zirconia by nurturing a stable tetragonal zirconia-yttrium oxide (mixed oxide) phase and shooting up the catalytic activity up to 70% H_2_ yield even against the prominent carbon deposition. Gadolinium promotional addition up to 4 wt % modifies the surface by formation of a cubic zirconium-gadolinium oxide phase, limits the size of NiO up to the lowest value of 7.5 nm, and makes reducible NiO-moderately interacted species available over the catalyst surface and resists the coke deposition. The 5Ni4Gd/Y + Zr catalyst showed about ∼80% constantly up to 24 h at 800 °C. The relative rise of the NiO crystallite size and the band gap of the 5Ni5Gd/Y + Zr catalyst (than 5Ni4Gd/Y + Zr) resulted in a relative drop of catalytic activity over 5Ni5Gd/Y + Zr (than 5Ni4Gd/Y + Zr).

## Supporting Information Available

SEM image and elemental analysis of the 5Ni4Gd/YZr and 5Ni5Gd/YZr catalysts; BET surface area, pore diameter, and pore volume over the 5Ni*x*Gd/Y + Zr (*x* = 0, 1, 2, 3, 4, 5) catalyst; catalyst characterization procedure; catalytic activity in terms of the H_2_ yield; CH_4_ conversion; CO yield and CO_2_ conversion with respect to time on stream over nonpromoted and Gd-promoted catalyst systems; XRD of fresh and spent 5Ni/Zr and 5Ni*x*Gd/Y + Zr (*x* = 0, 1, 2, 3, 4, 5) catalyst systems; H_2_-TPR of the 5Ni*x*Gd/Y + Zr (*x* = 1, 2, 3, 4) catalyst; H_2_-TPR → CO_2_-TPD → H_2_TPR cyclic experiment over 5Ni5Gd/Y + Zr; O_2_ temperature-programmed oxidation of the spent 5Ni4Gd/Y + Zr; RAMAN spectra of the 5Ni4Gd/Y + Zr catalyst; band gap of the 5Ni/Zr and 5Ni*x*Gd/YZr (*x* = 0, 1, 2, 3, 4, 5) catalysts.
