# Supplementary material for: Hydrogen Production from Gadolinium-Promoted Yttrium-Zirconium-Supported Ni Catalysts through Dry Methane Reforming
Source: ACS Omega. 2023 Jun 8;8(24):22108–20. doi: 10.1021/acsomega.3c02229 (PMC10286284; doi:10.1021/acsomega.3c02229)
Supplement: Supplementary file 1 — ao3c02229_si_001.pdf [file ao3c02229_si_001.pdf]

Hydrogen production from Gadolinium Promoted Yttrium-zirconium Supported Ni Catalyst through Dry Methane Reforming

Anis H. Fakeeha[1] , Ahmed S. Al-Fatesh[1]*, Vijay Kumar Srivastava,[2] Ahmed A. Ibrahim[1], Abdulaziz A.M. Abahussain[1], Jehad K. Abu-Dahrieh[3*], Mohammed F. Alotibi*[4], Rawesh Kumar[2]*

[1]Chemical Engineering Department, College of Engineering, King Saud University, P.O. Box 800, Riyadh, 11421, Saudi Arabia
[2]Department of Chemistry, Indus University, Ahmedabad, Gujarat, 382115, India
[3]School of Chemistry and Chemical Engineering, Queen's University Belfast, Belfast, BT9 5AG, Northern Ireland, UK
[4]Institute of Refining and Petrochemicals Technologies, King Abdulaziz City for Science and Technology (KACST), P.O. Box 6086, Riyadh 11442, Kingdom of Saudi Arabia

*Correspondence: j.abudahrieh@qub.ac.uk;aalfatesh@ksu.edu.sa; mfalotaibi@ksacst.edu.sa; kr.rawesh@gmail.com

Tel.: +44-28-9097-4603; +966-504158546

Support Information

(A)

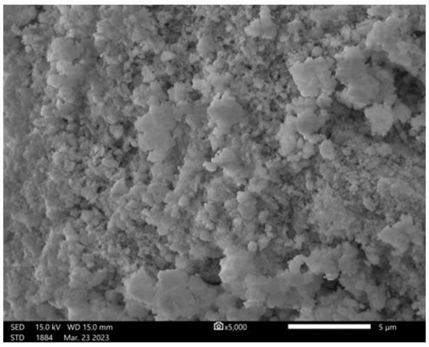

(B)

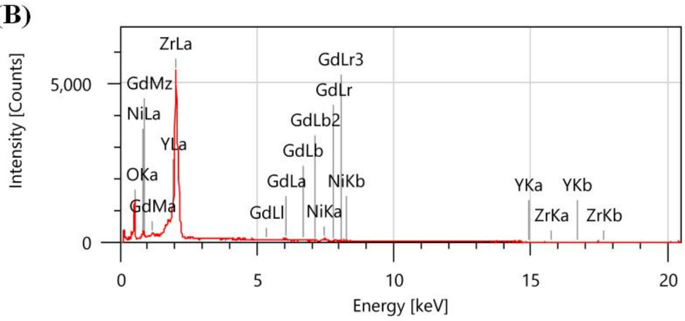

Element	Line	Mass%	Atom%
O	K	16.68±0.18	52.98±0.58
Ni	K	4.06±0.17	3.52±0.14
Y	L	9.09±0.25	5.19±0.15
Zr	L	66.90±0.37	37.26±0.21
Gd	L	3.27±0.19	1.06±0.06
Total		100.00	100.00

(C)

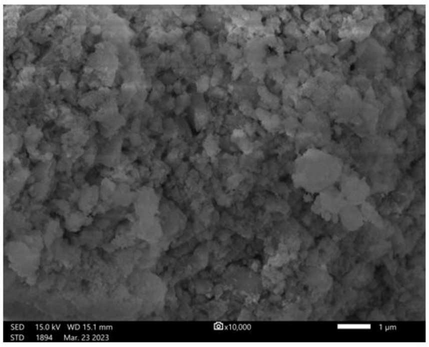

(D)

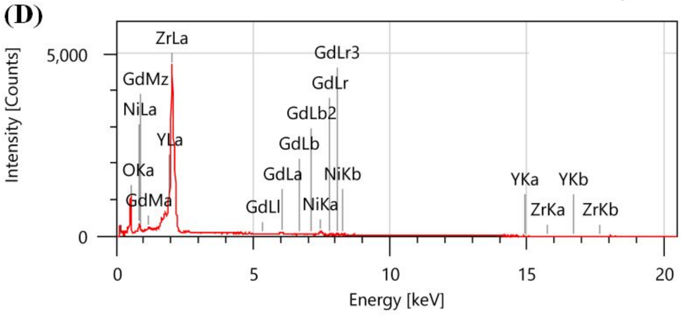

Element	Line	Mass%	Atom%
O	K	15.36±0.18	50.54±0.59
Ni	K	5.13±0.19	4.59±0.17
Y	L	9.89±0.27	5.85±0.16
Zr	L	64.87±0.38	37.42±0.22
Gd	L	4.76±0.22	1.59±0.07
Total		100.00	100.00

Figure S1. (A) SEM image 5Ni4Gd/YZr (B) EDX elemental analysis of 5Ni4Gd/YZr (C) SEM image 5Ni5Gd/YZr (D) EDX elemental analysis of 5Ni5Gd/YZr

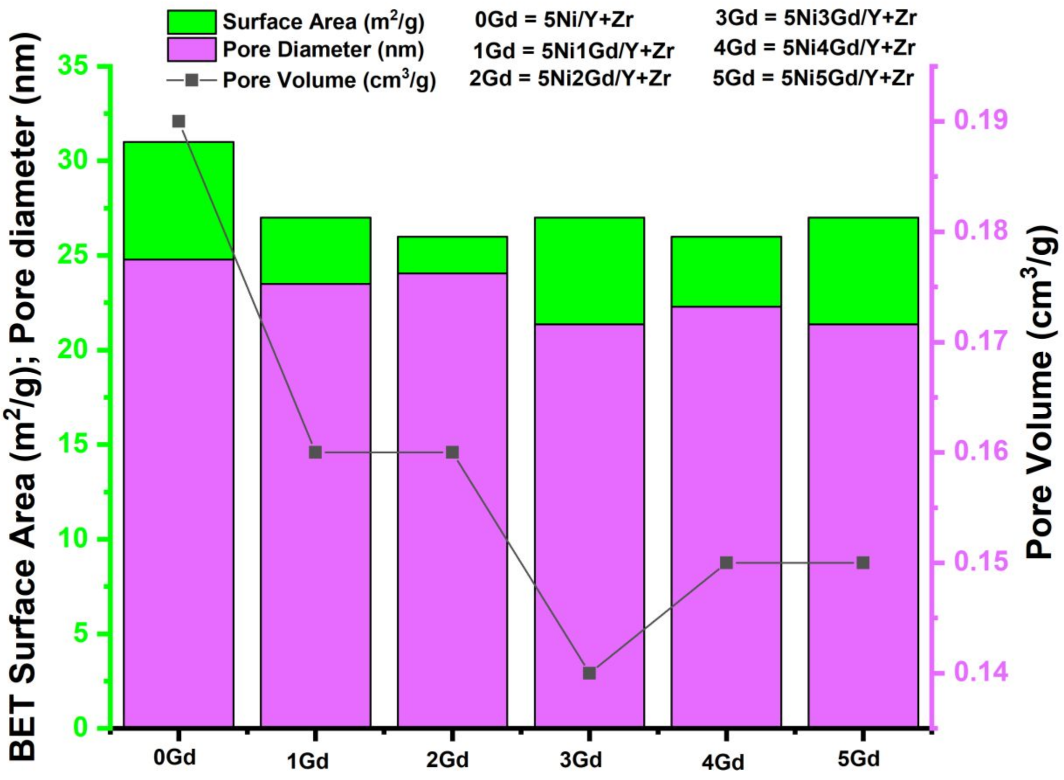

Figure S2. The BET surface area, Pore diameter and Pore volume over non-promoted and Gd promoted yttria-zirconia supported Ni catalyst.

Catalyst characterization

XRD :X-ray powder diffraction patterns for the samples were recorded on a Bruker D8 Advance (Bruker, Billerica, MA, USA) XRD diffractometer by using Cu K radiation source and a nickel filter, operated at 40 kV and 40 mA. The step size and scanning range of 2for analysis was set to 0.01 and 5–100, respectively. The present phases were documented using standard powder XRD cards (JCPDS).

Raman: "Laser Raman (NMR-4500) Spectrometer (JASCO, Japan) was used to obtain Raman spectra of the catalyst samples. The wavelength of the excitation beam was set to 532 nm and an objective lens of 100× magnification was used for the measurement. The laser intensity was adjusted to 1.6 mW for 10 seconds exposure time at 3 accumulations. This was to protect the sample from being damaged by laser irradiation. Measurement was done in the

range **150–1800** cm^{-1} (Raman shift) and the spectra were processed using Spectra Manager Ver.2 software (JASCO, Japan)."

IR: Fourier transform infrared (FTIR) spectroscopy was recorded employing IR Prestige-21 SHMADZU. The measurements were used to study the surface properties and functional groups in the samples.

UV: Ultraviolet spectroscopy (V-570, JASCO, USA) was used to study the optical properties of the samples. It uses a resolution of 1 nm in the range of 200 – 800 nm at a scanning speed of 200 nm/min.

TEM: The morphology of the catalysts was captured via 120 kV JEOL JEM-2100F transmission electron microscope (TEM).

H_2-TPR/CO_2-TPD

Automatic chemisorption equipment (Micromeritics Auto Chem II 2920, USA) was used to study temperature programmed reduction (TPR) and temperature programmed desorption (TPD) of the catalysts. 70 mg of the sample was subjected to a heat treatment for TPR at 10 °C/min up to 900 °C under atmospheric pressure and gas flow (40 ml/min) of 10% H_2/Ar or CH_4/Ar mixture. For TPD, 70 mg of sample was used. The sample was first kept at 200 °C for 1 h under helium flow to remove physically adsorbed species from the catalyst surface. Then, CO_2 adsorption was accomplished at 50 °C for 30 min by passing 10% CO_2/He mixture gas with a flow rate of 30 ml/min. Then, the CO_2 desorption signal was recorded by TCD with a linear increase in temperature up to 800 °C with a temperature ramp rate of 10 °C/min.

TGA: The amount of carbon deposition on the spent catalysts was assessed by thermal gravimetric analysis (TGA) under air by using a Shimadzu TGA-51.

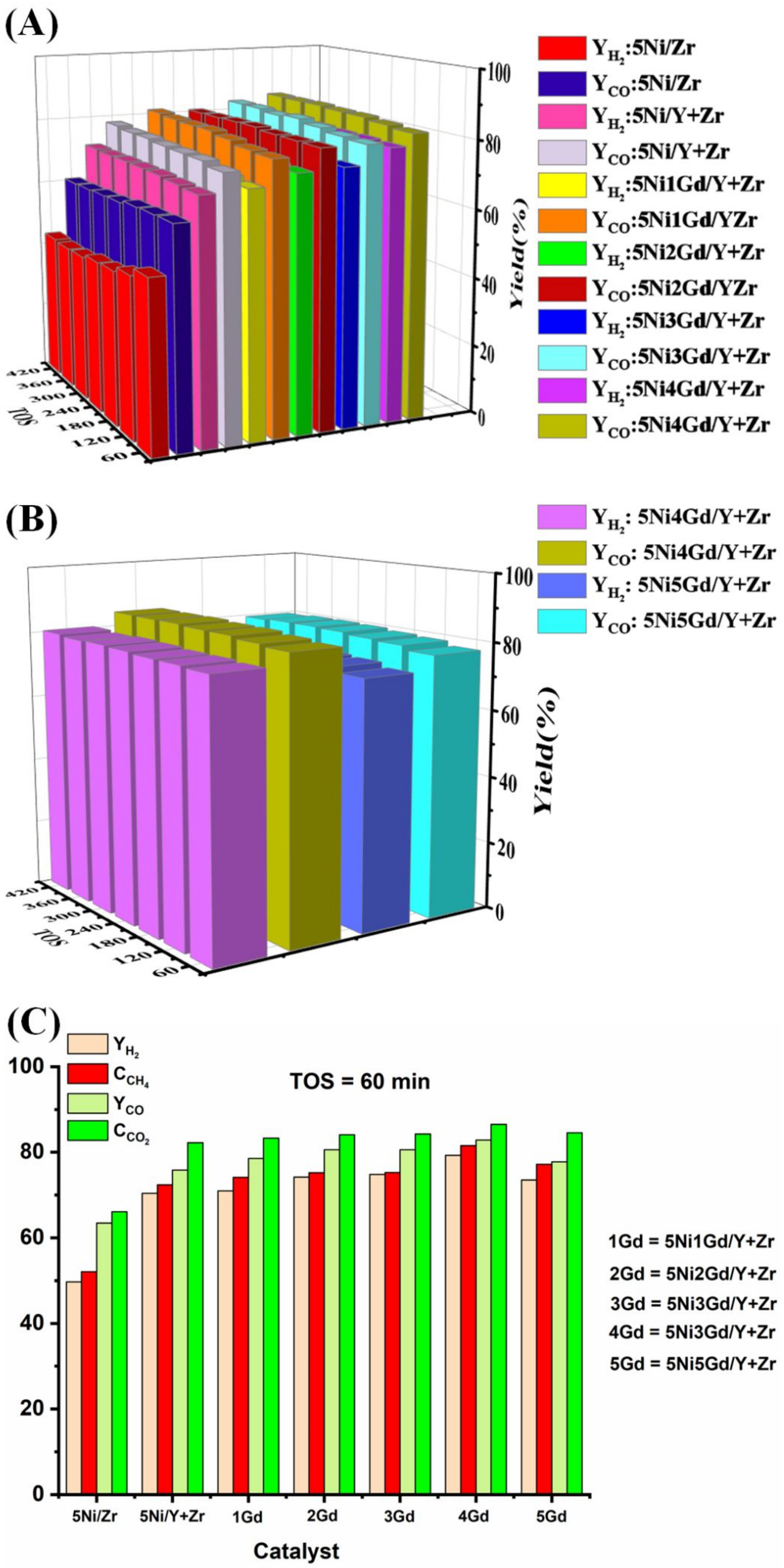
(A)
Y H2:5Ni/Zr
Y CO:5Ni/Zr
Y H2:5Ni/Y+Zr
Y CO:5Ni/Y+Zr
Y H2:5Ni1Gd/Y+Zr
Y CO:5Ni1Gd/YZr
Y H2:5Ni2Gd/Y+Zr
Y CO:5Ni2Gd/YZr
Y H2:5Ni3Gd/Y+Zr
Y CO:5Ni3Gd/Y+Zr
Y H2:5Ni4Gd/Y+Zr
Y CO:5Ni4Gd/Y+Zr
Yield(%)
0
20
40
60
80
100
TOS
60
120
180
240
300
360
420
(B)
Y H2: 5Ni4Gd/Y+Zr
Y CO: 5Ni4Gd/Y+Zr
Y H2: 5Ni5Gd/Y+Zr
Y CO: 5Ni5Gd/Y+Zr
Yield(%)
0
20
40
60
80
100
TOS
60
120
180
240
300
360
420
(C)
Y H2
C CH4
Y CO
C CO2
TOS = 60 min
0
20
40
60
80
100
5Ni/Zr
5Ni/Y+Zr
1Gd
2Gd
3Gd
4Gd
5Gd
Catalyst
1Gd = 5Ni1Gd/Y+Zr
2Gd = 5Ni2Gd/Y+Zr
3Gd = 5Ni3Gd/Y+Zr
4Gd = 5Ni3Gd/Y+Zr
5Gd = 5Ni5Gd/Y+Zr

Figure S3. (A) “H_2-yield and CO yield” vis-à-vis time on stream plot over 5Ni/Zr and 5NixGd/Y+Zr (x = 0, 1, 2, 3, 4) catalysts (B) “H_2-yield and CO yield” vis-à-vis time on stream plot over 5NixGd/Y+Zr (x = 4, 5) catalysts (C) H_2-Yield, CH_4 conversion, CO-yield and CO_2 conversion over 5Ni/Zr and 5NixGd/Y+Zr (x = 0, 1, 2, 3, 4) catalysts during 60-minute time on stream. Y_{H_2} = H_2-yield, C_{CH_4}= CH_4 conversion, Y_{CO} = CO-yield, C_{CO}= CO conversion

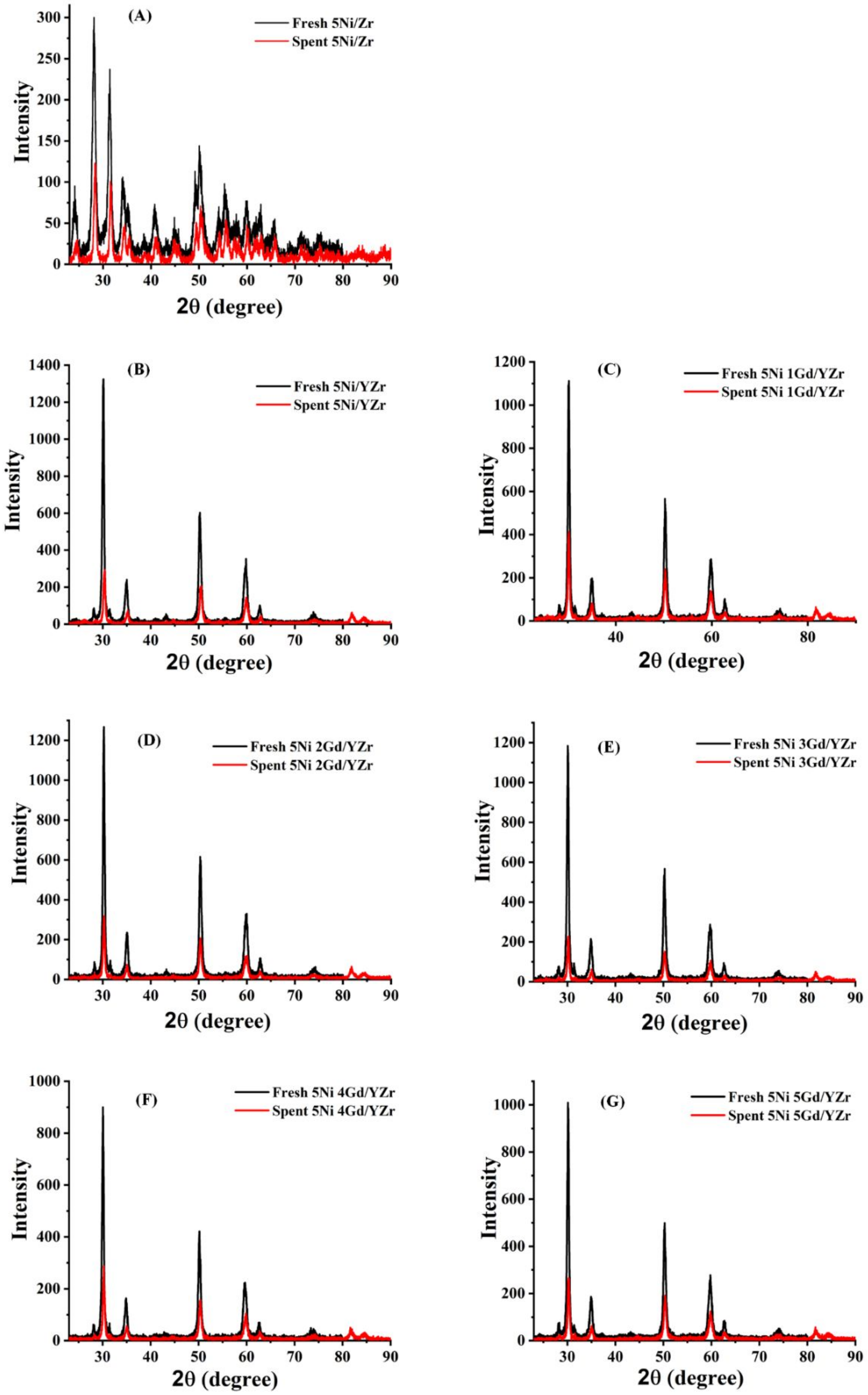

Figure S4. The XRD (A-G) of fresh vs spent of 5Ni/Zr and 5NixGd/Y+Zr (x = 0, 1, 2, 3, 4, 5) catalyst systems

Table S1. The bandgap of 5Ni/Zr and 5NixGd/YZr (x = 0, 1, 2, 3, 4, 5) catalysts

Catalyst Name	Fresh catalyst (bandgap)
5Ni/Zr	3.10
5Ni/Y+Zr	2.23
5Ni 1Gd/Y+Zr	3.30
5Ni 2Gd/Y+Zr	3.18
5Ni 3Gd/Y+Zr	3.18
5Ni 4Gd/Y+Zr	2.23
5Ni 5Gd/Y+Zr	3.00

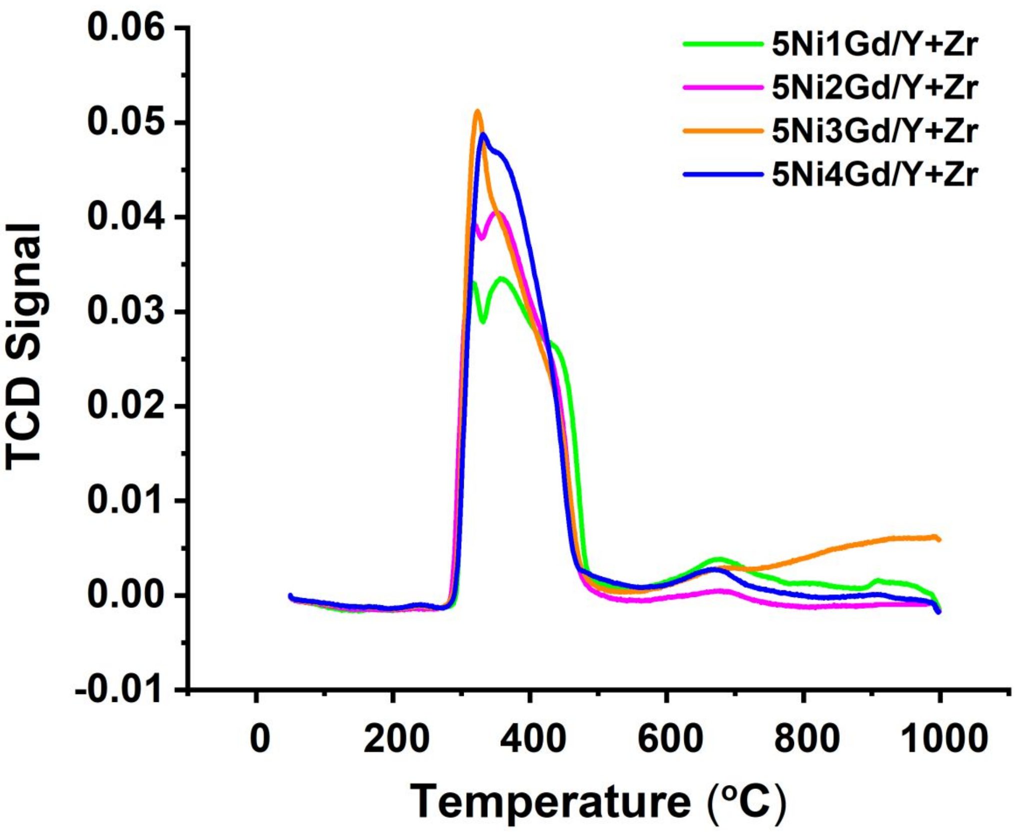

Figure S5. H_2-TPRof 5NixGd/Y+Zr (x = 1, 2, 3, 4) catalyst

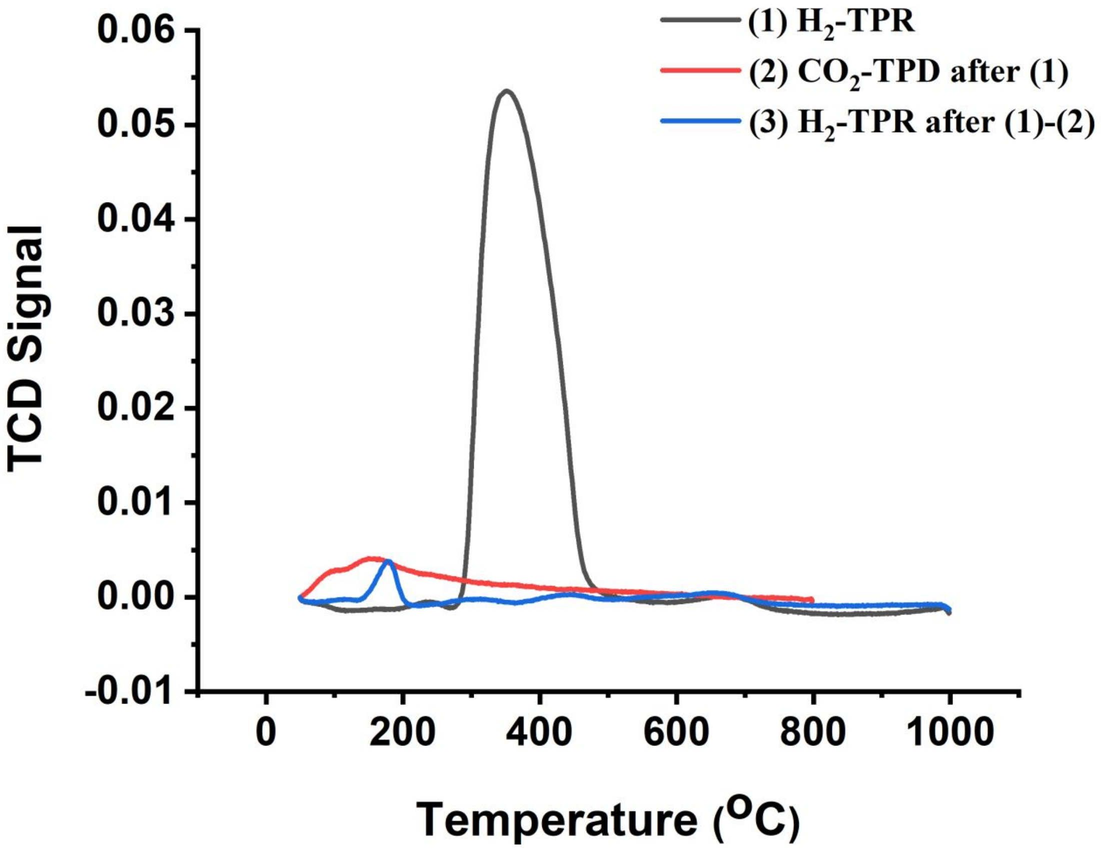

Figure S6. H_2-TPR→CO_2-TPD→H_2TPR cyclic experiment over 5Ni5Gd/Y+Zr

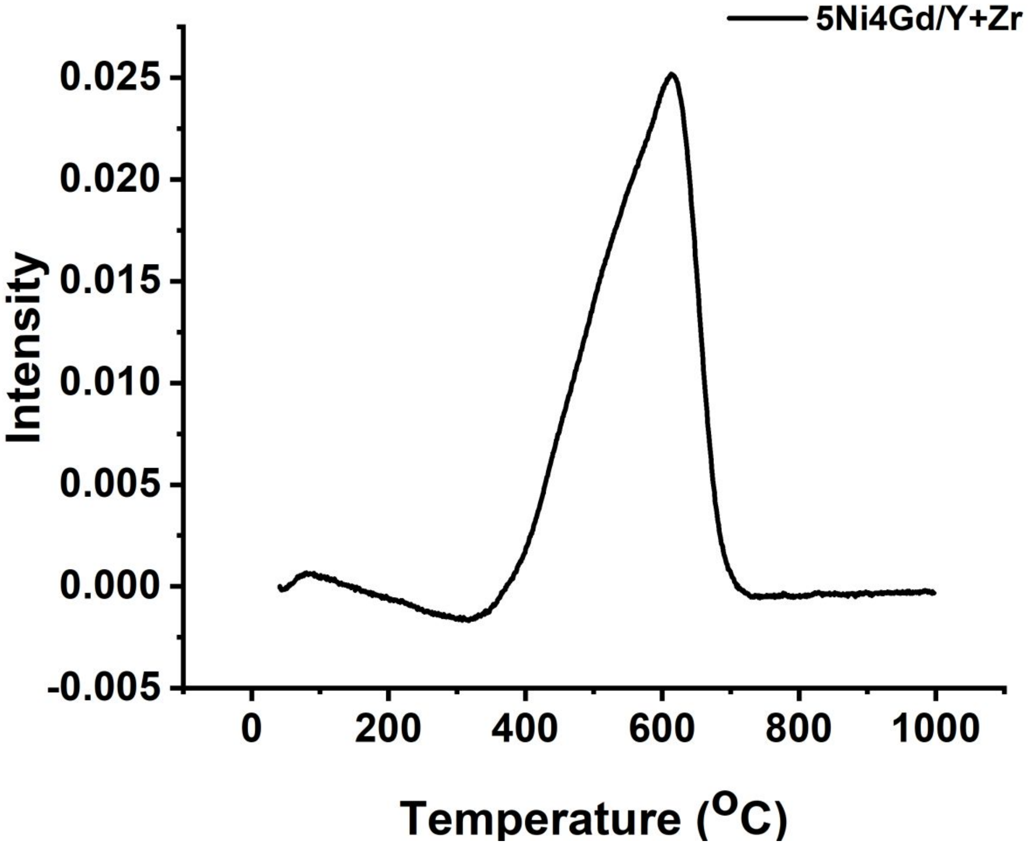

Figure S7. O_2-Temperature Programmed Oxidation of spent 5Ni4Gd/Y+Zr (collected after 24 h DRM reaction)

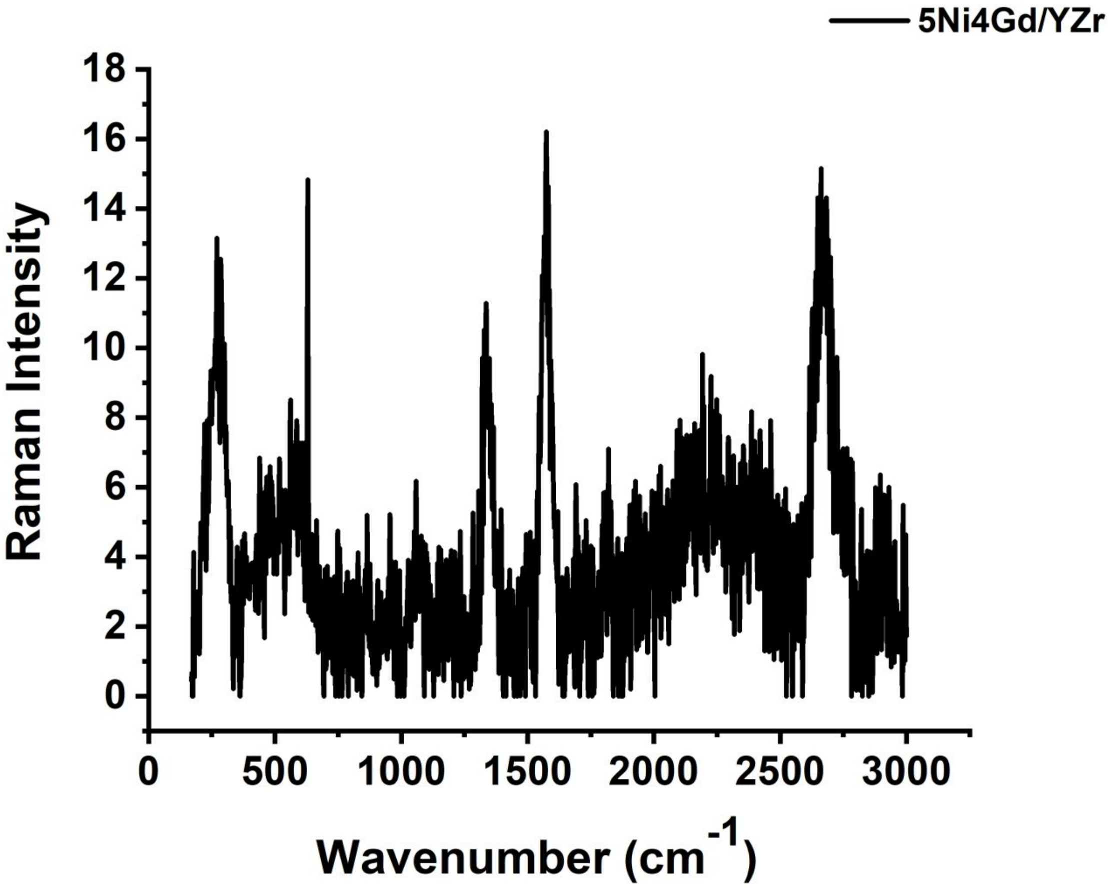

Figure S8. RAMAN spectra of 5Ni4Gd/Y+Zr catalyst
